# Large-Scale Dynamics of Self-propelled Particles Moving Through Obstacles: Model Derivation and Pattern Formation

**DOI:** 10.1007/s11538-020-00805-z

**Published:** 2020-09-25

**Authors:** P. Aceves-Sanchez, P. Degond, E. E. Keaveny, A. Manhart, S. Merino-Aceituno, D. Peurichard

**Affiliations:** 1 Department of Mathematics, North Carolina State University, Raleigh, NC 27695 USA; 2 Department of Mathematics, Imperial College London, London, SW7 2AZ UK; 3 Department of Mathematics, University College London, London, WC1H 0AY United Kingdom; 4 Faculty for Mathematics, University of Vienna, Oskar-Morgenstern-Platz 1, 1090 Vienna, Austria; 5 Department of Mathematics, University of Sussex, Brighton, BN1 9RH UK; 6 Inria Paris - MAMBA Project Team, Laboratory Jacques-Louis Lions, Sorbonne University, Paris, France

**Keywords:** Self-propelled particles, Hydrodynamic limit, Pattern formation, Stability analysis, Gradient flow, Non-local interactions, 35Q70, 82C05, 82C22, 82C70, 92B25, 92C35, 76S05

## Abstract

We model and study the patterns created through the interaction of collectively moving self-propelled particles (SPPs) and elastically tethered obstacles. Simulations of an individual-based model reveal at least three distinct large-scale patterns: travelling bands, trails and moving clusters. This motivates the derivation of a macroscopic partial differential equations model for the interactions between the self-propelled particles and the obstacles, for which we assume large tether stiffness. The result is a coupled system of nonlinear, non-local partial differential equations. Linear stability analysis shows that patterning is expected if the interactions are strong enough and allows for the predictions of pattern size from model parameters. The macroscopic equations reveal that the obstacle interactions induce short-ranged SPP aggregation, irrespective of whether obstacles and SPPs are attractive or repulsive.

**Electronic supplementary material:**

The online version of this article (10.1007/s11538-020-00805-z) contains supplementary material, which is available to authorized users.

## Introduction

This work is devoted to deriving and analysing a model of collectively moving self-propelled particles that interact with a complex, heterogeneous environment. The field of collective dynamics studies what happens when a large number of agents, which can be animals, people, micro-organisms, crystals, etc., interact with each other. A particular focus is the emergence of large-scale order or patterns. Famous examples include global alignment in crystals (de Gennes and Prost 1993), lane formation for people (Feliciani and Nishinari 2016), waves and aggregation in bacteria (Shimkets 1990; Ben-Jacob et al. 2000), milling in schools of fish (Shaw 1978) or swarming in birds (Cavagna et al. 2010). All these examples have in common that local, small-scale interaction rules between individuals lead to global, large-scale patterns. These patterns are typically hard or impossible to predict from the local interaction rules; hence, their understanding requires the use of either extensive simulations or mathematical analysis.

*Combining Collective Dynamics and Environmental Effects* In many systems, one also needs to take into account the environment to be able to explain observed patterns in collective phenomena (Chepizhko et al. 2013; Chepizhko and Peruani 2013; Jabbarzadeh et al. 2014; Park et al. 2008). For cells moving through a tissue, this environment often includes fibres and other components. For instance, it has been observed that many cell types have a tendency to move up stiffness gradients, a phenomenon termed *durotaxis* (Lo et al. 2000). In some of these instances, the effect on the substrate is negligible or the environment forms confining barriers affecting organism behaviour (Noselli et al. 2019). However, in many applications the interaction modifies the environment (either permanently or transiently) in a way that affects subsequent interactions. An example is the degradation of the extracellular matrix (ECM) caused by migrating cells (Baricos et al. 1995), which affects the ECM structure and hence future migration. In this work, we want to combine collectivity and environmental interactions and study the resulting patterns. Known examples of patterns created include travelling bands of large swarms of scavenging locust (Buhl et al. 2006; Topaz et al. 2008), the formation of paths in grass-land by active walkers (Helbing et al. 1997; Lam 1995) or aggregation of individuals (Bernoff and Topaz 2016). For metastasising cancer cells, it was observed that the invasion success depends on whether they move individually or as small clusters (Cheung and Ewald 2016).

*Obstacles Can Emulate Complex Environments* In this work, we focus on a particular type of environment in which objects interact with moving particles. Example applications include pedestrians avoiding obstacles (Helbing et al. 2005) or animal herds or fish swarms moving through vegetation. Our focus, however, lies on micro-scale applications, such as pathogens moving through visco-elastic tissue (Celli et al. 2009; Harman et al. 2012) or immune cells migrating through fibrous ECM (Baricos et al. 1995). The importance of the environment is particularly true for sperm dynamics, where the surrounding fluid plays a key role in the emergence of collective motion. For example, clustering and large-scale swirling was observed in simulations of collectively moving sperm in Schoeller and Keaveny (2018) and Sokolov et al. (2007). In Degond et al. (2019), a model was proposed that couples the Vicsek model for collective dynamics with Stokes equations for a viscous fluid. However, sperm dynamics takes place in a complex fluid, whose constitutive properties cannot be characterised solely by a viscosity. In Tung et al. (2017), it was reported that sperm moving through a visco-elastic fluid forms small clusters, a behaviour not observed in a purely viscous environment. To approximate the complex environment, the introduction of immersed obstacles has been proposed (Kamal and Keaveny 2018; Majmudar et al. 2012; Wróbel et al. 2016). For example, Kamal and Keaveny (2018) the authors propose a model in which an undulatory swimmer swims in a fluid filled with elastically tethered obstacles, however effects of collective dynamics, i.e. multiple swimmers, were not investigated. In this article, we present a model for collective motion in an environment filled with spheres tethered to fixed points in space via linear springs, that play the role of obstacles. We will study the impact of this obstacle-based environment on the collective dynamics for a large number of self-propelled particles (SPPs).

*Consequences for Aggregation* Aggregation of populations of individual animals or cells has been observed in many contexts and is usually attributed to individuals’ attraction (Parrish and Edelstein-Keshet 1999). A major finding of this work is that an initially homogeneous elastic environment can lead to particle aggregation in the absence of explicit attractive interactions between the particles themselves. Furthermore, we show that the obstacles can induce aggregation irrespective of whether they repel or attract the particles. This poses the question whether past conclusions about the cause of biological aggregation need to be reviewed: Some of the observed aggregative effect might have been caused by so far under-appreciated environmental interactions, rather than by direct interactions between individuals.

*Individual versus Continuum Description* From a modelling perspective, two approaches are common (Mogilner and Manhart 2016). One can formulate a system of individual-based models (IBMs), also called agent-based models, where the behaviour of each individual is assumed to be governed by separate, often stochastic ordinary differential equations. This approach has the advantage that the translation of modelling assumptions of the individual level is relatively straight-forward. However, few analytical tools are available to study IBMs and even if the system exhibits the desired property, limited insight can be gained as to why it does so. On the other hand, one can formulate a partial differential equation (PDE) model for the macroscopic quantities of interest, e.g. the space and time-dependent density of agents. A rich mathematical toolbox exists for the analysis of PDEs, which includes linear stability analysis, constructions of steady states as well as efficient simulation tools. Substantial progress has been made to establish systematic links between IBMs and the corresponding PDEs (Degond and Motsch 2008; Ha and Tadmor 2008). This allows to combine the advantages of both methods: straight forward translation of biological assumptions into the IBM and strong analytical tools for the PDE model. The self-organised hydrodynamics (SOH) approach (Degond and Motsch 2008) used in this work has been successfully applied, e.g. to fibre interactions (Peurichard 2016), bacterial swarms (Degond et al. 2018), sperm fertility (Creppy et al. 2016) or ant trail formation (Boissard et al. 2013).

*Paper Structure* In Sect. 2, we present the individual-based model, at whose basis lies the famous (Vicsek et al. 1995) model. This model describes SPPs that align their orientation with neighbouring particles, to which we add a short-ranged repulsion term. The environment consists of obstacles which are tethered via linear springs to anchor points fixed in space. SPPs and obstacles exert either repulsive or attractive forces on each other. Simulations of the IBM reveal the richness of possible patterns for this simple system, which includes clustering, trail formation and travelling bands, and motivate the formulation of a macroscopic PDE model of the SPP–obstacle interactions. The derivation of the macroscopic model, presented in Sect. 3, builds on the SOH technique for the SPPs, but requires new techniques for the obstacles. We focus on a particular asymptotic regime, where the obstacle tethering is strong, i.e. strong spring stiffness. The derived macroscopic model for SPP–obstacle interactions is presented and interpreted in Sect. 3.3 and the main theorem is proven in Sect. 3.4. We capitalise on the macroscopic model by analysing pattern formation through linear stability analysis in Sect. 4.1. In Sect. 4.2, we use the macroscopic model to discover that obstacles mediate an effective SPP interaction with biphasic behaviour. Finally in Sect. 5, we perform simulations in one space dimension of the macroscopic and individual-based model and compare the results to each other and the analytical results. In Sect. 6, we examine consequences of our findings for biology and discuss further steps.

**Fig. 1:**
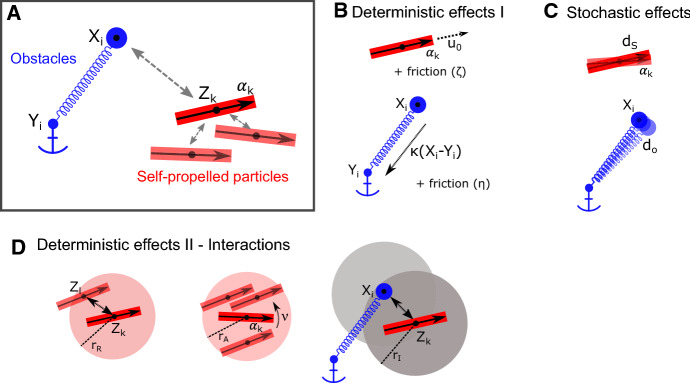
(Color figure online) Ingredients of the IBM. **a** Shown are the two types of agents of the IBM, the SPPs (red) and the obstacles (blue). **b** Deterministic effects that affect each agent individually. SPPs self-propel themselves and experience friction, obstacles are elastically tethered to their anchor points and also experience friction. **c** Stochastic effects for the SPPs (orientation) and the obstacles (position). **d** Interactions include SPP repulsion, SPP alignment and SPP–obstacle interactions

## The Individual-Based Model (IBM)

### Formulation of the IBM

The following model describes self-propelled individuals interacting with obstacles. Biologically, the particles in our model typically represent cells (such as cancer, sperm or bacterial cells), but can also describe animals or people. We assume they align and repel each other. Alignment might be an active behaviour (e.g. birds might try and adjust their movement direction to each other), or a passive effect caused, e.g. by the individuals’ shape (rod shaped bacteria might align upon collision (Peruani et al. 2006). Obstacles are elastically tethered to fixed anchor points. They mimic a complex environment, which acts on and reacts to particles, e.g. by repulsion. They can represent, e.g. a fibrous network which is relatively fixed in space, but whose components can be pushed upon interaction, after which they relax back towards their original position. Other applications could include plants such as trees or sea grass. In the following, we mathematically formalise these assumptions.

The starting point for our investigation is an individual-based model (IBM), in which the dynamics of each component is described by individual equations coupled through interaction terms. We couple the famous Vicsek model for collective movement of self-propelled particles (SPPs) (Vicsek et al. 1995) with an environmental model, described by elastically tethered obstacles. Our IBM is set in *n*-dimensional space, where $n=1,2$ or 3. The two components and interactions are depicted schematically in Fig. 1. Several applications of collective movement, in particular when applied to cells, take place at the micro-scale. These regimes are typically friction dominated with negligible inertia (also called over-damped regime). We therefore formulate our model in this friction dominated regime.

*Model Components* We model the following two types of agents:

- *Obstacles* We consider a set of *N* mobile obstacles with positions $X_i(t)\in {\mathbb {R}}^n$ for $i = 1, 2, \ldots , N$ and time $t\ge 0$. Each obstacle is tethered to a fixed anchor point $Y_i\in {\mathbb {R}}^n$ through a Hookean spring with stiffness constant $\kappa >0$ and experiences friction with the environment with friction constant $\eta >0$.
- *SPPs* We denote by $Z_k(t)\in {\mathbb {R}}^n$ the positions of the *k*-the SPP at time $t \ge 0$ for $k = 1, 2, \ldots , M$. Each SPP has a body orientation $\alpha _k(t)\in {\mathbb {S}}^{n-1}$ and a self-propulsion speed $u_0$ in direction $\alpha _k$. SPPs experience friction with the environment with friction constant $\zeta >0$.

*Interactions* We consider the following interactions:

- *SPP alignment* We assume each SPP aligns its body orientation $\alpha _k$ to the mean orientation ${{\bar{\alpha }}}_k$ of body directions of SPPs in its neighbourhood with radius $r_A$. This happens with an alignment frequency $\nu >0$ and is analogous to the famous Vicsek model for collective swarming (Vicsek et al. 1995).
- *SPP repulsion* SPPs repel each other at short distances, which models size-exclusion effects. Following Degond et al. (2015), we model this by an even pushing potential $\psi : {\mathbb {R}}^n \mapsto {\mathbb {R}}$ with typical spatial scale $r_R>0$. The force felt between two SPPs positioned at $Z_i$ and $Z_j$ is then given by $\nabla \psi \left( Z_i-Z_j\right)$.
- *Obstacle–SPP interaction* We assume the obstacles and SPPs exert a force on each other, which depends on the distance between them. Similar to the SPP repulsion, we describe this by an even interaction potential $\phi : {\mathbb {R}}^n \mapsto {\mathbb {R}}$ with typical scale $r_I$, yielding the force $\nabla \phi \left( Z-X\right)$ for a SPP at position *Z* and an obstacle at position *X*. In general, we assume this force to be repulsive; however, we will discuss the effect of an attractive force in Sect. 4.

*Stochasticity* We include two sources of uncertainty, both modelled by independent Brownian motions: Stochastic effects in the obstacle position (with intensity $d_o$) as well stochastic effects in the SPP orientation (intensity $d_s$).

*Model Equations* The effects described above can be modelled through the following coupled, stochastic ODEs. Note that in the absence of obstacles, the equations reduce to the time-continuous Vicsek model, described, e.g. in Vicsek et al. (1995). From here on, we work with the non-dimensional variables (but keeping the same names as introduced above), in particular we have chosen the domain size *L* as reference length and $L/u_0$ as reference time. The latter can be interpreted as the time it takes a freely moving SPP to cross the domain. We then obtain:

1a $$\begin{aligned} \,\mathrm{d}X_i =&-\frac{\kappa }{\eta }(X_i-Y_i)\,\mathrm{d}t -\frac{1}{\eta }\frac{1}{M}\sum _{ k = 1}^M \nabla \phi \left( X_i - Z_k\right) \,\mathrm{d}t+ \sqrt{ 2 d_o} \, \,\mathrm{d}B^{i}_t , \end{aligned}$$

1b $$\begin{aligned} \,\mathrm{d}Z_k =&\alpha _k \,\mathrm{d}t - \frac{1}{\zeta }\frac{1}{N}\sum _{i = 1}^N \nabla \phi \left( Z_k - X_i\right) \,\mathrm{d}t -\frac{1}{\zeta }\frac{1}{M} \sum _{l \ne k}^M \nabla \psi \left( Z_k - Z_l\right) \,\mathrm{d}t, \end{aligned}$$

1c $$\begin{aligned} \,\mathrm{d}\alpha _k =&P_{ \alpha _k^\perp } \circ \bigg [ \nu {\bar{\alpha }}_k \,\mathrm{d}t + \sqrt{2 d_s} \, \,\mathrm{d}{{\tilde{B}}}^{k}_t \bigg ], \end{aligned}$$

where the mean direction ${\bar{\alpha }}_k$ is defined via the mean flux $J_k$ by

2 $$\begin{aligned} {\bar{\alpha }}_k = \frac{ J_k}{ | J_k|},\quad \text { where } J_k =\sum _{\begin{array}{c} j = 1 \\ |Z_k-Z_j|\le r_A \end{array}}^M \alpha _j. \end{aligned}$$

The tether positions $Y_i$ are given and do not change in time. The operator $P_{\alpha _k^\perp }$ in (1c) is an orthogonal projection onto $\alpha _k^\perp$ and ensures that if $\alpha _k(0)\in {\mathbb {S}}^{n-1}$, then $\alpha _k(t)\in {\mathbb {S}}^{n-1}$ for all time. Note that we have scaled the interaction terms by the number of SPPs or obstacles to prepare for the kinetic limit of Sect. 3.1.

#### Remark 1

(Modelling choices) In an attempt to create a minimal model, we did not include a number of effects. For example, as opposed to Degond et al. (2015), we don’t model relaxation of the SPP orientation to the SPP velocity. Notice also that we did include repulsion between SPPs, but not repulsion between the obstacles. The former helps avoid collapse of the SPP density. For the obstacles on the other hand, this seems to be less likely due to their tethering in space. Also, there is no coupling to a surrounding fluid, which will be subject of future work.

### Simulations of the 2D IBM

We simulate the IBM (1) in two space dimensions. In this work, instead of doing a more thorough investigation, we want to showcase what types of patterns can be created based on the environmental interactions, emphasising the need for a PDE-based description.

*Simulation Set-Up* We simulate the IBM using $N=5000$ SPPs and $M=5000$ obstacles on a 2D square domain ${\mathcal {B}}=[0,1]\times [0,1]$ with periodic boundary conditions. We distribute the fixed anchor points $Y_i$ using a uniform distribution on ${\mathcal {B}}$ and initialise the obstacle positions with $X_i(0)=Y_i$. Initial SPP positions $Z_k(0)$ and orientations $\alpha _k(0)=(\cos (\varphi _k), \sin (\varphi _k))$ are both chosen at random with uniform distributions on ${\mathcal {B}}$ for $Z_k(0)$ and on $[0,2\pi ]$ for $\varphi _k$. For the interaction potentials, we use kernels of the following shape

$$\begin{aligned}&\phi (x)=\frac{3A_I}{2 r_I^3 \pi }(r_I-|x|)^2 H(r_I-|x|),\quad \psi (x)=\frac{3A_R}{2 r_R^3 \pi }(r_R-|x|)^2 H(r_R-|x|), \end{aligned}$$

where *H*(*x*) is the Heaviside function. These kernels are compactly supported on balls with radius $r_I$ and $r_R$, respectively, and chosen to yield a continuous pushing force decreasing linearly. They are normalised such that the force mass is $A_I$ and $A_R$, respectively. For simplicity, we choose all interaction radii to be the same, i.e. $r_I=r_A=r_R$. We leave the following parameters constant: $d_o=0$, $\eta =1$, $d_s=0.1$, $A_I=1$. We’re left with five parameters: $\kappa$, $\zeta$, $\nu$, $r_I$ and $A_R$.

**Fig. 2:**
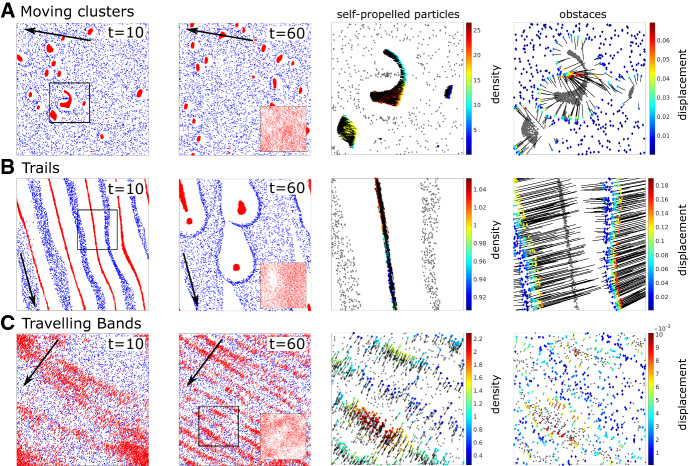
(Color figure online) IBM patterns. Depicted are snapshots for three different example patterns from the IBM simulations: Moving clusters (**a**), trails (**b**) and travelling bands (**c**). The first two columns show the SPPs (red arrows) and the obstacles (blue diamonds) in the full 2D simulation domain at two different time points, black arrows mark the mean SPP direction. Insets in the second column show resulting SPP positions for simulations without obstacles. The last two columns show enlargements of the black box in the first columns. In the ‘SPP’ column, the obstacles are shown in grey and the SPPs as black arrows, colors mark SPP neighbourhood density. In the ‘obstacles’ column, SPPs are shown in grey. The lines connect each obstacle to their anchor point, color marks obstacle displacement. Videos can be found in the Supp. Mat

**Fig. 3:**
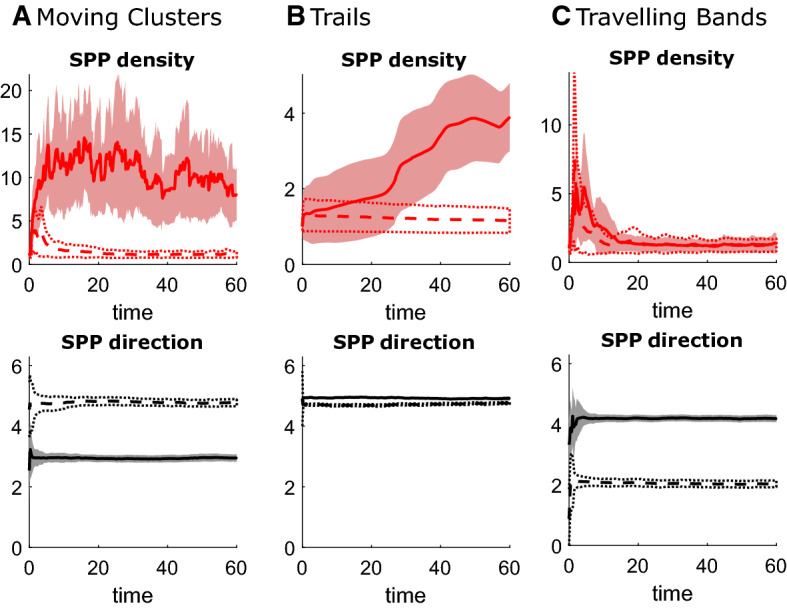
(Color figure online) IBM statistics. Shown are some statistics for the IBM patterns in Fig. 2 for moving clusters (**a**), trails (**b**) and travelling bands (**c**). Solid and dashed lines mark averages for simulations with and without obstacles, respectively, shaded areas and dotted lines corresponding averages ± standard deviations. SPP densities are calculated for each SPP by calculating the density within a disc of radius $r_A=r_R$ and dividing by the mean density in the domain

*IBM Simulation Results* Figure 2 shows examples of the different patterns produced by different choices of parameters, and Fig. 3 shows some associated statistics. Corresponding videos can be found in Supp. Mat. The second row in Fig. 3 shows that in all three cases SPPs globally align, i.e. the variance in SPP direction decreases. We call the observed patterns: *Moving clusters* ($\kappa =100$, $\zeta =1$, $\nu =10$, $r_I=0.05$, $A_R=0.01$), *Trails* ($\kappa =2.5$, $\zeta =10$, $\nu =100$, $r_I=0.15$, $A_R=0.002$) and *Travelling bands* ($\kappa =100$, $\zeta =40$, $\nu =10$, $r_I=0.05$, $A_R=0.002$) and give a short description of them.

*Moving Clusters* In this regime, tether stiffness and SPP–obstacle repulsion are high. The SPPs form very high density groups moving through the obstacles, whose displacement from the anchor points is relatively low. In Fig. 2a, we see how a larger cluster is split into two due to the obstacles, suggesting that the cluster size is controlled by the dynamics. This might also be the reason for the relatively large changes in mean SPP density over time seen in Fig. 3. Nevertheless, this pattern seems to be stable.

*Trails* Here, SPP alignment is strong, with low tether stiffness. The SPPs form stripes parallel to their movement direction, which at $t=10$ seem to be very regularly spaced. Within the stripes, the SPPs are close together and consequently push the obstacles away from the trails, leading to large obstacle displacements. Interestingly, the trails become unstable and by $t=60$, the SPPs form moving groups. We see this instability building up and the trails falling apart around $t=26$ in Fig. 3b. The enlargements in Fig. 2 indicate that the instability of the trails might stem from the fact that the obstacles are not symmetrically displaced to the right and left of the moving trails.

*Travelling Bands* In this pattern, the spring strength is high and obstacle displacements are consequently small. SPPs now form bands normal to their direction of movement. At $t=60$, we see in Fig. 2 that there appears to be a typical spacing between the bands. These patterns seem to be stable.

*Obstacles Reinforce and Diversify Patterns* To assess the influence of the environment on the pattern formation, we compare to simulations of the model without obstacles, i.e. pure Vicsek-type dynamics with small SPP repulsion. In the inset in the second column in Fig. 2, we see that in all three examples there is no patterning in absence of the obstacles. Figure 3 shows that the alignment behaviour seems unaltered by the obstacles, however for moving clusters and trails the obstacles lead to much higher SPP densities. It is known that for some specific ranges of parameters, clusters and bands already appear in simulations of the Vicsek model alone (Vicsek et al. 1995). However, in the presence of obstacles their qualitative behaviour is different: the environment seems to reinforce such structures and the travelling bands appear to be regularly spaced, which is not the case for bands in the Vicsek model alone. In addition, the homogeneous phase (common to the Vicsek dynamics) appears to be less common here. Finally, we observe that also a completely new pattern emerges: trails.

*The Need for a PDE Description* The three patterns found by simulating the IBM show that the interactions between SPPs and obstacles can lead to a rich repertoire of patterns such as clustering, trails and travelling bands. While the system is relatively simple, the number of parameters (about 15) make it prohibitively expensive to explore fully the complete parameter space: Total computation time with an 1.80GHz Intel Core i7 CPU was on the order of magnitudes of hours for one simulation shown in Fig. 2. Simulation time increases with the number of SPPs and obstacles, with larger interaction radii as well as with larger forces produced by the dynamics (e.g., by clustering), which necessitates smaller time steps. The shown patterns were found by rough and preliminary parameter scans and we expect that there exist in fact many more patterns. For each example pattern, a number of questions arise:

- *Clusters* It seems that large clusters split, leading to an intrinsic cluster size. If that is the case how is cluster size controlled and how is it determined from parameters? What determines particle density inside clusters?
- *Trails* The observed trails appear to be a transient, unstable pattern. Is the instability due to the asymmetry in obstacle displacement and what causes it? Can other parameters produce stable trails?
- *Travelling bands* Is there a set band wavelength and if yes, what determines it? When are these patterns stable?

All these questions suggest that a continuous, PDE-based description of the system is crucial to understanding the observed patterns, as well as to discover others. A PDE-description has several advantages: Patterns such as travelling bands can be constructed explicitly and a stability analysis can performed. Further the PDE description is inherently an averaging process reducing the number of parameters. Lastly, since instead of numerically solving thousands of coupled ODEs, one has to solve only a few PDEs, simulations become much more efficient. The next section is therefore devoted to the derivation of the PDE-based description of the SPP–obstacle model.

## Derivation of the Macro-model

**Fig. 4:**
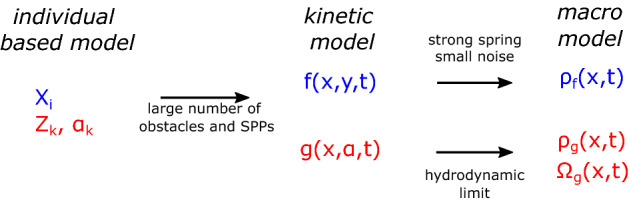
(Color figure online) Derivation overview. Shown are the different levels of models used in this work and the connection between them. See text for further details

In this section, we derive a macroscopic PDE-based model for the SPPs and the obstacles. The IBM model in (1) serves as the starting point. The derivation is a two-step process: First we formally derive a kinetic description for both the SPPs and the obstacles by taking a mean-field limit. In the second step, we use a hydrodynamic scaling for the SPPs and derive equations for the SPP density and orientation. For this step, we use previous work (Degond and Motsch 2008; Degond et al. 2015). For the obstacles, we focus on a particular parameter regime and assume to have low obstacle noise and strong obstacle spring stiffness. The main technical difficulty and new derivation strategy lie in this last step. Figure 4 summarises the different derivation steps. Throughout the document, the domains of integrations are understood to mean the whole domain, unless specified otherwise.

### The Mean-Field Limit

We start by defining $g( x, \alpha ,t)$, the distribution of the SPPs at position $x \in {\mathbb {R}}^n$, time $t \ge 0$ with direction of the self-propelled velocity $\alpha \in {\mathbb {S}}^{n-1}$ and let *f*(*x*, *y*, *t*) be the distribution of obstacles with position $x \in {\mathbb {R}}^n$, tethered at $y \in {\mathbb {R}}^n$ at time $t \ge 0$.

We consider the empirical distribution associated with the dynamics of the SPPs and tethered obstacles given by system (1).

3 $$\begin{aligned} g^M ( x, \alpha , t)&= \frac{ 1}{ M} \sum _{k=1}^M \delta _{Z_k(t)} ( x) \otimes \delta _{ \alpha _k(t)} ( \alpha ) \, , \nonumber \\ f^N ( x, y, t)&= \frac{ 1}{ N} \sum _{i=1}^N \delta _{X_i(t)} ( x) \otimes \delta _{ Y_i(t)} ( y), \end{aligned}$$

where $\delta _{A}$ denotes the Dirac delta in ${\mathbb {R}}^n$ (for $A=X_i, Y_i, Z_k$) or in ${\mathbb {S}}^{n-1}$ (for $A=\alpha _k$) concentrated at *A*.

#### Lemma 1

(Kinetic Model) Formally, as $N, M \rightarrow \infty$, $f^N \rightarrow f$ and $g^M \rightarrow g$, where the distributions *f*(*x*, *y*, *t*) and $g(x,\alpha ,t)$ fulfil the following Kolmogorov–Fokker–Planck equations

4a $$\begin{aligned}&\partial _tf + \nabla _x \cdot \big ( {\mathcal {W}} f \big ) = d_o \varDelta _x f, \end{aligned}$$

4b $$\begin{aligned}&\partial _tg + \nabla _x \cdot \big ( {\mathcal {U}} g \big ) + \nabla _\alpha \cdot \bigg ( P_{\alpha ^\perp } \left[ \nu {\bar{\alpha }}_g \right] g \bigg ) = d_s \varDelta _\alpha g, \end{aligned}$$

where

5 $$\begin{aligned}&{\bar{\alpha }}_g(x,t) = \frac{ J_g ( x, t)}{| J_g ( x, t)|} \qquad \text { with} \qquad J_g ( x, t) = \int _{|x-z|\le r_A}\!\!\! \alpha \, g ( z, \alpha , t) \,\mathrm{d}z \,\mathrm{d}\alpha . \end{aligned}$$

For the (space and time dependent) velocities, we have

6 $$\begin{aligned} {\mathcal {W}}&= - \frac{\kappa }{\eta } ( x - y) - \frac{1}{\eta } \nabla _x {\bar{\rho }}_g(x,t),\nonumber \\ {\mathcal {U}}&= \alpha - \frac{ 1}{ \zeta } \nabla _x {\bar{\rho }}_f(x,t)- \frac{ 1}{\zeta } \nabla _x \breve{\rho }_g(x,t), \end{aligned}$$

where we have introduced the densities of obstacles and SPPs

7 $$\begin{aligned} \rho _g(x,t)=\int g ( x, \alpha , t) \,\mathrm{d}\alpha ,\qquad \rho _f(x,t)=\int f( x, y, t) \,\mathrm{d}y, \end{aligned}$$

as well as an abbreviation for densities convoluted with kernels

$$\begin{aligned} {{\bar{\rho }}}(x,t):=(\phi * \rho )(x,t), \qquad \breve{\rho }(x,t):=(\psi * \rho )(x,t). \end{aligned}$$

Further *f* fulfils

8 $$\begin{aligned} \int f(x,y,t) \,\mathrm{d}x = \rho _A(y), \end{aligned}$$

where $\rho _A (y)$ is a given, time-independent function of obstacle anchor positions.

#### Proof

The limit is purely formal and uses standard techniques. We observe that $f^N$ and $g^M$ fulfil the equations for all *N* and *M* and then pass to the limit. $\square$

#### Remark 2

Note that since *f* and *g* are probabilities, they also fulfil

$$\begin{aligned} \int f(x,y,t) \,\mathrm{d}x \,\mathrm{d}y = \int g(x, \alpha , t) \,\mathrm{d}x \,\mathrm{d}\alpha \equiv 1, \end{aligned}$$

and consequently

$$\begin{aligned} \int \rho _A (y) \,\mathrm{d}y = 1. \end{aligned}$$

*Interpretation* At this point, we have a system of coupled kinetic equations for the obstacle distribution *f*(*x*, *y*, *t*) and the SPP distribution $g(x,\alpha ,t)$. The interactions between the obstacles and the SPPs lead to the terms of the form $\nabla _x{{\bar{\rho }}}$ in the speeds ${\mathcal {W}}$ and ${\mathcal {U}}$ in (6). An easy way to understand these terms is by assuming that the interaction force is of repulsive nature and purely local, in which case $\nabla _x{{\bar{\rho }}}=\nabla _x \rho$. We then see that the interaction force moves obstacles and SPPs in the opposite direction of the gradient of each other. The convolution with $\phi$ accounts for the potential non-locality of this interaction, which will be crucial later on. The remaining terms in ${\mathcal {W}}$ and ${\mathcal {U}}$ show the influence of the tethers and the self-propulsion for obstacles and the SPPs, respectively. In ${\mathcal {U}}$, we also see the influence of SPP repulsion. The term involving $\nabla _\alpha$ in (4b) reflects the effect of SPP alignment. The terms on the right-hand side of (4) are results of the stochasticity in the obstacle position (for *f*) and in the SPP orientation (for *g*).

### Scaling Assumptions

To derive the macroscopic equations for the SPP–obstacle interactions, we make a number of scaling assumptions for both the SPPs and the obstacles.

*Scaling Assumptions for the SPPs* Following previous work Degond and Motsch (2008), Degond et al. (2015), we introduce a small parameter $\varepsilon$ and specify the relative order of the various terms. We mostly follow Degond et al. (2015), with a few small differences: Firstly we assume the effect of alignment to be purely local, i.e. $r_A={\mathcal {O}}(\varepsilon )$, as has been done e.g. in Degond and Motsch (2008). Alternatively one could choose a weakly non-local scaling $r_A={\mathcal {O}}(\sqrt{\varepsilon })$, which would lead to an additional viscous term in the SPP orientation equation (13b) below. As in Degond et al. (2015), we also assume the SPP self-repulsion to be purely local, i.e. $r_R={\mathcal {O}}(\varepsilon )$ and assume that

$$\begin{aligned} \int \psi (x)\,\mathrm{d}x=:\mu <\infty . \end{aligned}$$

However, we do not make any smallness assumption with regard to the SPP–obstacle interaction scale $r_I$. This is because we are interested in studying the effect of the non-locality of this interaction. Otherwise we proceed as in Degond et al. (2015), i.e. assuming the alignment frequency $\nu$ and orientational diffusion $d_s$ to be of order $1/\varepsilon$, and their ratio to be of order one.

*Scaling Assumptions for the Obstacles* From (6), we see that it is only the macroscopic obstacle density $\rho _f(x,t)$ that enters the SPP equation. Unfortunately, we cannot obtain a closed system for the macroscopic obstacle density $\rho _f(x,t)$ of *f* by integrating (4a). Instead we make assumptions about the time scales of the obstacle dynamics. From now on, we also assume to have a constant anchor density, i.e. $\rho _A \equiv 1$ is constant in space and time. We note that the results can be generalised to non-uniform $\rho _A$. We introduce the following quantities

$$\begin{aligned} \gamma =\eta /\kappa ,\qquad \delta =d_o \gamma . \end{aligned}$$

For the derivation, we will assume both $\gamma$ and $\delta$ to be small. For $\gamma$, this means that the obstacle spring relaxation time scale is small compared to the SPP domain crossing time. We will sometimes refer to this assumption as ‘stiff obstacles’, since it can be realised with a large spring constant $\kappa$. For $\delta$, smallness means that the obstacle spring relaxation time scale is small compared to the obstacle diffusion time scale, which we refer to as ‘low obstacle noise’. Next we rewrite (4a) as

9 $$\begin{aligned} \partial _tf + \nabla _x \cdot \left( {{\tilde{v}}}(x,t) f\right) =\frac{1}{\gamma } {\mathcal {A}}_y(f), \end{aligned}$$

where we have defined the ‘external’ velocity as

10 $$\begin{aligned} {{\tilde{v}}}(x,t)=-\frac{1}{\eta }\nabla _x{\bar{\rho }}_g(x,t) \end{aligned}$$

and the operator ${\mathcal {A}}_y$ by

11 $$\begin{aligned} {\mathcal {A}}_y(f):=\nabla _x \cdot \left[ (x-y)f + \delta \nabla _x f \right] . \end{aligned}$$

We can rewrite the operator as

$$\begin{aligned} {\mathcal {A}}_y(f)=\delta \nabla _x \cdot \left[ M_\delta (x-y)\nabla _x\left( \frac{f}{M_\delta (x-y)}\right) \right] , \end{aligned}$$

where $M_{\delta }(z)$ is a Gaussian with variance $\delta$ centred around 0, whose mass is normalised to one, i.e.

12 $$\begin{aligned} M_{\delta }(z)=\frac{1}{Z_\delta }e^{-\frac{|z|^2}{2\delta }}, \qquad Z_\delta =(2\pi \delta )^{n/2}. \end{aligned}$$

The above also shows that $M_\delta (x-y)$ is in the kernel of ${\mathcal {A}}_y$.

#### Remark 3

Note that the rescaling of the diffusion term $\delta =d_o\gamma$ ensures the operator ${\mathcal {A}}_y$ is a Fokker–Planck-type operator. Without it, we would obtain ${\mathcal {A}}_y(f)=\nabla _x \cdot \left[ (x-y)f\right]$, whose kernel contains Dirac deltas, making the analysis much more tedious. Eventually, however, we are interested in the small noise limit. This, of course raises several questions, which are beyond the scope of this work, e.g. does the order of the limits $\gamma \rightarrow 0$ and $\delta \rightarrow 0$ matter?

### The Macroscopic SPP–Obstacle Equation

Using the scaling and notation above, we now state the main result of this section, which we prove in Sect. 3.4.

#### Theorem 1

(SPP–Obstacle Macromodel) Let $\rho _A\equiv 1$ be constant and *f*(*x*, *y*, *t*) fulfill (9) with $\gamma \ll 1$ and $\delta \ll 1$. Further let $g^\varepsilon (x,\alpha ,t)$ be the solution of (4b) using the scaling involving $\varepsilon$ described above and let $g^0(x,\alpha ,t)$ be its (formal) limit as $\varepsilon \rightarrow 0$. Then it holds that

$$\begin{aligned} g^0(x,\alpha ,t)=\rho _g(x,t)N_{\Omega _g(x,t)}(\alpha ), \end{aligned}$$

where $N_{\Omega }$ is the von Mises–Fisher distribution defined by

$$\begin{aligned} N_{\Omega }(\alpha )=\frac{1}{K_{d}}e^{\frac{\Omega \cdot \alpha }{d}},\qquad K_{d}=\int e^{\frac{\Omega \cdot \alpha }{d}} \,\mathrm{d}\alpha , \qquad d=\frac{d_s}{\nu }, \qquad \text {for}\quad \Omega \in {\mathbb {S}}^{n-1}. \end{aligned}$$

Note that $K_d$ is a normalisation constant and is independent of $\Omega$. Further the macroscopic SPP density $\rho _g(x,t)$ and the macroscopic SPP orientation $\Omega _g(x,t)$ fulfil

13a $$\begin{aligned}&\partial _t\rho _g + \nabla _x \cdot \left( U \rho _g \right) = 0, \end{aligned}$$

13b $$\begin{aligned}&\rho _g \partial _t\Omega _g + \rho _g \left( V \cdot \nabla _x\right) \Omega _g + d P_{\Omega _g^\perp } \nabla _x \rho _g = 0, \end{aligned}$$

13c $$\begin{aligned}&U=c_1\Omega _g-\frac{1}{\zeta }\nabla _x {\bar{\rho }}_f-\frac{\mu }{\zeta }\nabla _x \rho _g, \quad V=c_2\Omega _g-\frac{1}{\zeta }\nabla _x {\bar{\rho }}_f-\frac{\mu }{\zeta }\nabla _x \rho _g, \end{aligned}$$

The constants $c_1>0$ and $c_2>0$ depend only on $d=d_s/\nu$ and are defined as in Degond et al. (2015). The macroscopic obstacle density $\rho _f(x,t)$ is given by

14 $$\begin{aligned} \rho _f(x,t)=1&- \frac{\gamma }{\delta \eta } \bigg [ {\bar{\rho }}_g(x)-\big [M_{2\delta }*{\bar{\rho }}_g\big ](x) \bigg ] \nonumber \\&-\frac{\gamma ^2}{\eta }\partial _t \varDelta _x {{\bar{\rho }}}_g +\frac{\gamma ^2}{\eta ^2}{\mathcal {N}}({{\bar{\rho }}}_g)+{\mathcal {O}}(\gamma ^2\delta )++{\mathcal {O}}(\gamma ^3), \end{aligned}$$

where the nonlinear term ${\mathcal {N}}$ is defined by

$$\begin{aligned} {\mathcal {N}}({{\bar{\rho }}}_g)=\frac{1}{2}\left[ (\varDelta _x \bar{\rho }_g)^2-{\mathbb {H}}({{\bar{\rho }}}_g):{\mathbb {H}}({{\bar{\rho }}}_g)\right] \, , \end{aligned}$$

where ${\mathbb {H}}({{\bar{\rho }}}_g)$ denotes the Hessian of the function ${{\bar{\rho }}}_g$, i.e. $\{ {\mathbb {H}}({{\bar{\rho }}}_g)\}_{i,j} = \partial _i \partial _j {{\bar{\rho }}}_g$, and given two *n* by *n* matrices ${\mathbb {A}}$ and ${\mathbb {B}}$, their scalar product is defined as ${\mathbb {A}}:{\mathbb {B}} = \sum _{i,j=1}^n A_{i,j} B_{i,j}$.

Equations (13a) and (13b) give the evolution for the particle density $\rho _g$ and mean orientation $\Omega _g$, respectively. Without the term $\nabla _x{{\bar{\rho }}}_f$ appearing in *U* and *V* in Eq. (13c), these equations correspond to the so-called Self-Organised Hydrodynamics with Repulsion (SOHR) and their derivation can be found in Degond et al. (2015). The additional terms in Eq. (13c) account for the influence of the obstacles density $\rho _f$.

The equation for the obstacle density, expanded in the small variables $\delta$ and $\gamma$, is given in (14). It is important to note that the obstacle density given in (14) can in principle become negative, which is not physically meaningful. This is a consequence of the assumption that $\gamma$ is small and indicates that the validity of the model will be limited to certain parameter regimes. We see that for infinitely strong springs, i.e. $\gamma \rightarrow 0$, $\rho _f(x,t)\equiv \rho _A\equiv 1$, i.e. obstacles remain exactly at their anchor points and since those are assumed to be uniformly distributed, the obstacles have no effect on the SPPs ($\nabla _x{{\bar{\rho }}}_f\equiv 0$). For small, but finite $\gamma$ the feedback from the SPPs leads to non-uniform obstacles.

*Influence of Obstacle Noise* The influence of the obstacle noise $\delta$ is contained in the order $\gamma$ term in (14). We note that

$$\begin{aligned} - \frac{1}{\delta \eta } \bigg [ {\bar{\rho }}_g(x)-\left( M_{2\delta }*{\bar{\rho }}_g\right) (x) \bigg ]\rightarrow \frac{1}{\eta }\varDelta _x {\bar{\rho }}_g(x)\qquad \text {as}\quad \delta \rightarrow 0. \end{aligned}$$

We see that the noise adds an additional form of non-locality. Whether the obstacle density is reduced or increased depends on whether ${\bar{\rho }}_g$, the convoluted SPP density at *x* is smaller or larger than the ‘blurred’, convoluted SPP density ${\bar{\rho }}_g$, where the amount of blurring depends on the obstacle noise. In the absence of obstacle noise, (14) simplifies to

15 $$\begin{aligned} \rho _f(x,t)=1 +\frac{\gamma }{\eta }\varDelta _x {\bar{\rho }}_g(x)-\frac{\gamma ^2}{\eta }\partial _t \varDelta _x {{\bar{\rho }}}_g +\frac{\gamma ^2}{\eta ^2}{\mathcal {N}}({{\bar{\rho }}}_g)+{\mathcal {O}}\big (\gamma ^3\big ). \end{aligned}$$

*SPP Dynamics Deform Obstacle Volume Elements*  In the absence of obstacle noise, we can rewrite (15) as

16 $$\begin{aligned} \rho _{f}(x)&=\det {J_Y}-\frac{\gamma ^2}{\eta }\partial _t \varDelta _x \bar{\rho }_g+{\mathcal {O}}\big (\gamma ^3\big ), \end{aligned}$$

where $J_Y$ is the Jacobian of the map

$$\begin{aligned}&Y(x,t)=x+\frac{\gamma }{\eta }\nabla _x{\bar{\rho }}_g(x,t). \end{aligned}$$

The map *Y* can be interpreted as an estimate of the anchor position of an obstacle at position *x* moved under the influence of the SPP density. Then the determinant of the Jacobian reflects the deformation of a volume element of obstacles due to the SPPs. Note that for $n=3$ $\det {J_Y}$ contains also order $\gamma ^3$ terms, for $n=2$ only order $\gamma ^2$ terms and lower.

*Higher-Order Terms Account for SPP Movement*  Finally, we comment on the time derivative appearing in (14). The time derivative leads to a form of delay, i.e. the obstacles retain a memory of where SPPs were. This can be seen by Taylor expanding the SPP density in time using the time scale of obstacle relaxation $\gamma$. Then the linear terms in (15) can be written as

$$\begin{aligned} \frac{\gamma }{\eta }\varDelta _x\left( {{\bar{\rho }}}_g-\gamma \partial _t{{\bar{\rho }}}_g\right) =\frac{\gamma }{\eta }\varDelta _x {{\bar{\rho }}}_g(x,t-\gamma )+{\mathcal {O}}\big (\gamma ^2\big ). \end{aligned}$$

Finally in preparation for the analytical and numerical investigation of Sects. 4 and 5, we state the following:

#### Corollary 1

(1D equations.) Let the assumptions of Theorem. 1 hold. Then for $n=1$, the equations for the SPP density $\rho _g(x,t)$ and the obstacle density $\rho _f(x,t)$ with $x\in {\mathbb {R}}$ and $t\ge 0$ are given by

17 $$\begin{aligned}&\partial _t\rho _g+c_1\partial _x \rho _g=\frac{1}{\zeta }\partial _x\left( \mu \rho _g\partial _x\rho _g+\rho _g\partial _x {{\bar{\rho }}}_f\right) , \end{aligned}$$

where we have assumed all particles move to the right. The obstacle density up to order $\gamma ^2$ is given by

18 $$\begin{aligned} \rho _f(x,t)=1&- \frac{\gamma }{\delta \eta } \bigg [ {\bar{\rho }}_g(x)-\big [M_{2\delta }*{\bar{\rho }}_g\big ](x) \bigg ]-\frac{\gamma ^2}{\eta }\partial _t \partial _x^2 {{\bar{\rho }}}_g. \end{aligned}$$

For $\delta \rightarrow 0$ and using only terms up to order $\gamma$, (18) simplifies to

19 $$\begin{aligned} \rho _f(x,t)=1 + \frac{\gamma }{\eta }\partial _x^2{{\bar{\rho }}}_g. \end{aligned}$$

### Proof of Theorem 1

For the coarse-graining of the kinetic SPP equation (4b), we refer to previous work (Degond and Motsch 2008; Degond et al. 2015). We note that the obstacle density enters the SPP equation solely through its macroscopic density $\rho _f(x,t)$ via the interaction operator $\nabla _x{\bar{\rho }}_f$, which has a structure analogous to the SPP self-repulsion term, hence analogous techniques can be applied.

To derive an expression for the obstacle density $\rho _f(x,t)$, we formulate and prove the following Theorem:

#### Theorem 2

Let $\rho _A\equiv 1$ and *f*(*x*, *y*, *t*) fulfil (9) with ${\mathcal {A}}_y(f)$ defined in (11). Let $\gamma \ll 1$ and expand *f*(*x*, *y*, *t*) as

20 $$\begin{aligned} f(x,y,t)=f_0(x,y,t)+\gamma f_1(x,y,t)+\gamma ^2 f_2(x,y,t)+{\mathcal {O}}(\gamma ^3). \end{aligned}$$

Then the macroscopic densities defined by

21 $$\begin{aligned} \rho _{f_i}(x,t)=\int f_i(x,y,t) \,\mathrm{d}y \end{aligned}$$

satisfy

22 $$\begin{aligned} \rho _{f_0}(x)&=1\nonumber \\ \rho _{f_1}(x)&=-\mathrm{div} ({{\tilde{v}}})-\delta \frac{1}{2}\varDelta _x \mathrm{div} ({{\tilde{v}}})+ {\mathcal {O}}(\delta ^2),\nonumber \\ \rho _{f_2}(x)&=\frac{1}{2}\nabla _x \cdot \left[ {{\tilde{v}}}\, \mathrm{div}{({{\tilde{v}}})}-({{\tilde{v}}}\cdot \nabla _x){{\tilde{v}}}\right] +\partial _t \mathrm{div}{({{\tilde{v}}})}+ {\mathcal {O}}(\delta ), \end{aligned}$$

as $\delta \rightarrow 0$. We use the notation $\text {div}= \nabla _x \cdot$ for the divergence of a vector field.

#### Proof

In the following, we drop the *t*-dependence of most terms to increase readability. We obtain the following equations for the three highest orders of $\gamma$

23a $$\begin{aligned}&{\mathcal {A}}_y(f_0)=0, \end{aligned}$$

23b $$\begin{aligned}&{\mathcal {A}}_y(f_1)=\partial _tf_0+\nabla _x \cdot ({{\tilde{v}}}(x)f_0), \end{aligned}$$

23c $$\begin{aligned}&{\mathcal {A}}_y(f_2)=\partial _tf_1 +\nabla _x \cdot ({{\tilde{v}}}(x)f_1). \end{aligned}$$

Let us note that (23a), (23b), and (23c) can be recast as follows: Given a function *h* find $\psi$ (in a suitable functional space) such that

24 $$\begin{aligned} {\mathcal {A}}_y( \psi ) = h \, . \end{aligned}$$

Due to the conservation of mass property of ${\mathcal {A}}_y$, i.e. $\int {\mathcal {A}}_y \,\mathrm{d}x = 0$, a necessary condition to warranty the existence of a solution of (24) is $\int h \,\mathrm{d}x = 0$. It can be shown that the operator ${\mathcal {A}}_y$ has compact resolvent on a suitable functional space and its kernel is generated by $M_{\delta }(x-y)$, given in (12). The most important properties of the Gaussian $M_\delta$, that we will use repeatedly are

$$\begin{aligned} \int M_{\delta }(z) \,\mathrm{d}z=1, \qquad \int z M_{\delta }(z) \,\mathrm{d}z=0, \quad \nabla _z M_{\delta }(z)=-\frac{z}{\delta }M_{\delta }(z). \end{aligned}$$

Hence, we can obtain a complete characterisation of the solutions of (24) via the Fredholm alternative, namely, for any function *h* such that $\int h \,\mathrm{d}x = 0$ there exists a unique solution $\psi$ up to an element of the kernel of ${\mathcal {A}}_y$. For a proof of this result, consult (Aceves-Sanchez et al. 2019).

Let us start by considering (23a), we search for a solution $f_0$ such that $\int f_0 ( x, y) \,\mathrm{d}x = 1$; hence, according to the results obtained for (24), the unique solution is given as

25 $$\begin{aligned} f_0(x,y)= M_{\delta }(x-y), \end{aligned}$$

where $M_{\delta }$ is defined in (12). For the remaining two equations, we require the following scaling condition to hold, which ensures that the average mass is one,

26 $$\begin{aligned} \int f_i(x,y,t)\,\mathrm{d}x=0,\qquad i=1,2. \end{aligned}$$

*Step 1: Rescaling* Next we define the functions $h_1(\sigma , y,t)$ and $h_2(\sigma , y,t)$ as

$$\begin{aligned} f_1(x,y,t)&=\frac{1}{\sqrt{\delta }}\, M_\delta (x-y)\,h_1\left( \frac{x-y}{\sqrt{\delta }},y,t\right) ,\\ f_2(x,y,t)&=\frac{1}{\delta }\, M_\delta (x-y)\,h_2\left( \frac{x-y}{\sqrt{\delta }},y,t\right) . \end{aligned}$$

This turns (23b) and (23c) into equations for $h_1(\sigma ,y,t)$ and $h_2(\sigma ,y,t)$. Defining ${\mathcal {B}}$ as the operator

27 $$\begin{aligned}&{\mathcal {B}}(h)=\varDelta _\sigma h-\sigma \cdot \nabla _\sigma h, \end{aligned}$$

we obtain, after tedious but straightforward computations, the following relationships

28 $$\begin{aligned} {\mathcal {B}}(h_1)&=\sqrt{\delta }\,\text {div}({{\tilde{v}}})|_{y+\sqrt{\delta }\sigma }-\sigma \cdot {{\tilde{v}}}|_{y+\sqrt{\delta }\sigma },\nonumber \\ {\mathcal {B}}(h_2)&=\sqrt{\delta }\left( \partial _th_1 + h_1\,\text {div}({{\tilde{v}}})|_{y+\sqrt{\delta }\sigma }\right) +{{\tilde{v}}}|_{y+\sqrt{\delta }\sigma }\cdot (\nabla _\sigma h_1-\sigma \cdot h_1). \end{aligned}$$

There are several advantages to this scaling: Firstly, the operator ${\mathcal {B}}$ is the generator of the Ornstein–Uhlenbeck stochastic process (a consequence of using $\sigma =(x-y)/\sqrt{\delta }$) and we can use its well-known properties directly without having to scale by $\delta$. Secondly, we have removed the Gaussian $M_{\delta }$ from the equation (it cancelled). Finally, additionally scaling $f_1$ and $f_2$ by $1/\sqrt{\delta }$ and $1/\delta$, respectively, turns out to be the correct choice when calculating the densities.

Before we proceed to the next step, we need to collect a number of properties of ${\mathcal {B}}$, all of which are well known and stated in “Appendix A.2”.

*Step 2: Expansion in terms of the obstacle noise* $\delta$. The next step involves expansion of the right-hand sides of (28), $h_1$ and $h_2$ with respect to $\delta$, i.e.

$$\begin{aligned} h_1(\sigma ,y,t)&=h_1^0(\sigma ,y,t)+\sqrt{\delta }h_1^1(\sigma ,y,t)+\delta h_1^2(\sigma ,y,t)+{\mathcal {O}}(\delta ^{3/2}),\\ h_2(\sigma ,y,t)&=h_2^0(\sigma ,y,t)+\sqrt{\delta }h_2^1(\sigma ,y,t)+\delta h_2^2(\sigma ,y,t)+{\mathcal {O}}(\delta ^{3/2}). \end{aligned}$$

This yields as equations for $h_1^0$, $h_1^1$ and $h_1^2$

$$\begin{aligned} {\mathcal {B}}\big (h_1^0\big )&=-{{\tilde{v}}}_k \sigma _k,\\ {\mathcal {B}}\big (h_1^1\big )&=\partial _i {{\tilde{v}}}_i- \sigma _k \sigma _i \partial _k {{\tilde{v}}}_i, \\ {\mathcal {B}}\big (h_1^2\big )&=\sigma _k \partial _{ki}{{\tilde{v}}}_i - \frac{1}{2}\sigma _k \sigma _i \sigma _j \partial _{ij}{{\tilde{v}}}_k. \end{aligned}$$

Note that we have used the Einstein’s summation convention and that now ${{\tilde{v}}}$ and its derivatives are all evaluated at (*y*, *t*). Here partial derivatives are understood to act on the spatial variable, i.e. $\partial _i {{\tilde{v}}}:= \frac{\partial }{\partial y_i} {{\tilde{v}}}(y,t)$. The advantage of this procedure is the following: Now the right-hand sides are low-order polynomials in $\sigma$ and since ${\mathcal {B}}$ only acts on $\sigma$, the equations can be solved explicitly by rewriting the right-hand sides in terms of the Hermite basis and using P2 of Lemma 4 in “Appendix A.2”. This procedure yields the explicit solutions

29 $$\begin{aligned} h_1^0(\sigma ,y,t)&={{\tilde{v}}}_i{\mathcal {H}}_{e_i},\nonumber \\ h_1^1(\sigma ,y,t)&=\frac{1}{2}\partial _k {{\tilde{v}}}_j {\mathcal {H}}_{e_k+e_j},\nonumber \\ h_1^2(\sigma ,y,t)&=\frac{1}{2}\left[ \partial _{ii}{{\tilde{v}}}_k\, {\mathcal {H}}_{e_k}+\frac{1}{3}\partial _{ij}{{\tilde{v}}}_k\, {\mathcal {H}}_{e_k+e_i+e_j}\right] , \end{aligned}$$

where ${\mathcal {H}}$ are the tensor Hermite polynomials defined in Lemma 4 in “Appendix A.2”. Note that ${{\tilde{v}}}$ and all its derivatives are evaluated at (*y*, *t*) and ${\mathcal {H}}$ at $\sigma$.

As equations for $h_2^0$ and $h_2^1$, we obtain

$$\begin{aligned} {\mathcal {B}}(h_2^0)&={{\tilde{v}}}\cdot \left( \nabla _\sigma h_1^0-\sigma h_1^0\right) ,\\ {\mathcal {B}}(h_2^1)&=\partial _th_1^0+\partial _i {{\tilde{v}}}_i\,h_1^0 +(\sigma _k\partial _k{{\tilde{v}}}) \cdot \left( \nabla _\sigma h_1^0-\sigma h_1^0\right) + {{\tilde{v}}}\cdot \left( \nabla _\sigma h_1^1-\sigma h_1^1\right) . \end{aligned}$$

As above ${{\tilde{v}}}$ and its derivatives are all evaluated at (*y*, *t*). Using the solutions for $h_1^0$, $h_1^1$ and $h_1^2$ given in (29), we can solve the equations for $h_2^0$ and $h_2^1$ in the same fashion, yielding the explicit expressions

30 $$\begin{aligned} h_2^0(\sigma ,y,t)&=\frac{1}{2}{{\tilde{v}}}_k{{\tilde{v}}}_j {\mathcal {H}}_{e_k+e_j},\nonumber \\ h_2^1(\sigma ,y,t)&=\left( -\partial _t{{\tilde{v}}}_k + {{\tilde{v}}}_i \partial _i {{\tilde{v}}}_k\right) \, {\mathcal {H}}_{e_k}+\frac{1}{2}{{\tilde{v}}}_i\partial _k{{\tilde{v}}}_j\, {\mathcal {H}}_{e_k+e_i+e_j}. \end{aligned}$$

Note that the solutions fulfil the scaling condition (26) since it holds that

31 $$\begin{aligned} \int M_1(\sigma )h_i^j(\sigma ,y,t)\,\mathrm{d}\sigma =0,\quad i=1,2,\quad j=0,1,2. \end{aligned}$$

*Step 3: Calculating the macroscopic moments of the obstacle density* With the preparation of the two steps above, the calculation of the obstacle densities

$$\begin{aligned} \rho _{f_i}(x,t)=\int f_i(x,y,t) \,\mathrm{d}y, \end{aligned}$$

and consequently its contribution to the SPP equation becomes relatively simple. The procedure and calculations are described in “Appendix A.3”. This yields (22) as claimed. $\square$

*Explicit Solution for* $f_1$ The above outlined procedure works for any given external velocity ${{\tilde{v}}}(x,t)$, i.e. it allows to include other influences as well. For example, in future work we plan to use the derivation strategy to include the description of a fluid in which the obstacles and SPPs are immersed in. However, for this model, we can use the fact that ${{\tilde{v}}}(x,t)$ is in fact a conservative vector field. This allows to solve the first-order equation (23b) for $f_1(x,y,t)$ directly. This is covered in the following lemma, where the *t* dependence has been suppressed for notational convenience.

#### Lemma 2

Let ${{\tilde{v}}}(x)$ be a conservative vector field, i.e. there exists a scalar function *V*(*x*), such that $\nabla _x V={{\tilde{v}}}$, then we can write the solution to (23b) as

$$\begin{aligned} f_1(x,y)=M_{\delta }(x-y)\frac{1}{\delta }\left[ V(x)-\left( M_{\delta }*V\right) (y)\right] \end{aligned}$$

#### Proof

By direct calculation, we see that

$$\begin{aligned}&(x-y)f_1+\delta \nabla _x f_1 = M_\delta (x-y){{\tilde{v}}}(x), \end{aligned}$$

which shows that $f_1$ is indeed a solution to (23b). Finally, we have to verify the normalisation condition (26)

$$\begin{aligned} \int f_1(x,y) \,\mathrm{d}x&=\frac{1}{\delta }\int M_{\delta }(x-y)\left[ V(x)-\left( M_{\delta }*V\right) (y)\right] \,\mathrm{d}x\\&=\frac{1}{\delta }\left[ \int M_{\delta }(x-y)V(x)\,\mathrm{d}x-\left( M_{\delta }*V\right) (y)\right] =0, \end{aligned}$$

which finishes the proof. $\square$

The above Lemma is applicable for this model of SPP–obstacle interactions since we have that

$$\begin{aligned} {{\tilde{v}}}(x,t)=-\frac{1}{\eta }\nabla _x {\bar{\rho }}_g(x,t), \end{aligned}$$

i.e. we can use Lemma 2 with $V(x,t)=-\frac{1}{\eta } {\bar{\rho }}_g(x,t)$. We consequently find

$$\begin{aligned} f_1(x,y,t)&=- M_{\delta }(x-y)\frac{1}{\delta \eta }\left[ {\bar{\rho }}_g(x,t)-(M_{\delta }*{\bar{\rho }}_g)(y,t)\right] . \end{aligned}$$

From this, we can calculate

32 $$\begin{aligned} \rho _{f_1}(x,t)&=- \frac{1}{\delta \eta } \bigg [ {\bar{\rho }}_g(x)-\left( M_{2\delta }*{\bar{\rho }}_g\right) (x) \bigg ]. \end{aligned}$$

#### Remark 4

Note that since

$$\begin{aligned} - \frac{1}{\delta \eta } \bigg [ {\bar{\rho }}_g(x)-\left( M_{2\delta }*{\bar{\rho }}_g\right) (x) \bigg ]&=\frac{1}{\eta }\left[ \varDelta _x {\bar{\rho }}_g(x)+\frac{\delta }{2}\varDelta _x^2 {\bar{\rho }}_g\right] +{\mathcal {O}}(\delta ^2), \end{aligned}$$

we see that this is consistent with (22), but contains more information about the ${\mathcal {O}}(\delta ^2)$ term.

*The Macroscopic Obstacle Density* Collecting the results of Theorem 2 and Lemma 2 and using the definition of ${{\tilde{v}}}$ given in (10), we find that the maximum order of approximation of the obstacle density we can now write is given in (14) as claimed. This finishes the proof of Theorem 1. $\square$

## Analytical Insights from the 1D Macromodel.

In this section, we analyse the macroscopic model derived in Sect. 3 further to gain insights into the SPP–obstacle interactions. In particular, we use linear stability analysis to understand the onset of patterning and investigate how obstacles induce an effective SPP interaction.

### Linear Stability Analysis

In this section, we investigate pattern formation for the SPP–obstacle model. We work in one space dimension, i.e. we focus on the SPP density $\rho _g(x,t)$ and the obstacle density $\rho _f(x,t)$ for $x\in {\mathbb {R}}$ or and $t\ge 0$, whose dynamics are given by (17) and (18).

Consider the steady-state solution $\rho _g(x,t)=\rho _0>0$. Small perturbations of this solutions (called again $\rho _g$) then fulfil the linearised equation

33 $$\begin{aligned}&\partial _t\rho _g + c_1 \partial _x \rho _g = \frac{\rho _0}{\zeta } \left( \mu \partial ^2_x \rho _g+\partial ^2_x {\bar{\rho }}_f \right) , \end{aligned}$$

where $\rho _f$ is still given by (18).

The following propositions examine the growth or decay behaviour of perturbations of the constant steady state in dependence on their angular frequency and the resulting linear stability of the constant steady state. We consider the equation on the whole space $x\in {\mathbb {R}}$ and posed on an interval with periodic boundary conditions.

#### Proposition 1

(Linear stability) Consider (33) coupled to (18) posed (a) on $x\in {\mathbb {R}}$ and (b) on $x\in [0,1]$ with periodic boundary conditions.

(i) The system permits solutions of the form $\rho _g(x,t)=\rho e^{i k x + \alpha t}$, with $\rho \ne 0$, $\alpha \in {\mathbb {C}}$ and $k\in {\mathbb {R}}$ (case a) or $k\in 2\pi {\mathbb {Z}}$ (case b) where $\alpha$ and *k* fulfil the following dispersion relation

34 $$\begin{aligned} \alpha (k) = -i \frac{k c_1}{1+\gamma ^2\frac{\rho _0}{\eta \zeta }k^2 {{\hat{\phi }}}_k^2}+\frac{\rho _0}{\zeta }k^2 \frac{\frac{\gamma }{\eta \delta }\left( 1-e^{-\delta k^2}\right) {{\hat{\phi }}}_k^2-\mu }{1+\gamma ^2\frac{\rho _0}{\eta \zeta }k^2 {{\hat{\phi }}}_k^2}, \end{aligned}$$

where ${{\hat{\phi }}}_k$ is the Fourier transform (case a) or Fourier coefficient (case b) of the kernel $\phi$, defined by

$$\begin{aligned} {{\hat{\phi }}}_k=\int e^{-ikx}\phi (x) \,\mathrm{d}x, \end{aligned}$$

where the integration domain is understood to be ${\mathbb {R}}$ (case a) or [0, 1] (case b).

(ii) The constant steady state $\rho _g(x,t)=\rho _0$ is linearly stable iff

35 $$\begin{aligned} \max _{k\in K} \frac{1}{\delta }\left( 1-e^{-\delta k^2}\right) {{\hat{\phi }}}_k^2<\frac{\mu \eta }{\gamma }, \end{aligned}$$

where $K={\mathbb {R}}$ (case a) or $K=2\pi {\mathbb {Z}}$ (case b).

#### Proof

We show the proof for case a, case b can be shown analogously. (i) Substituting the ansatz $\rho _g(x,t)=\rho e^{k i x + \alpha t}$ into (33) is equivalent to applying the Fourier transform to the whole equation. We use the following properties of the Fourier transform

$$\begin{aligned} \widehat{f*g}={{\hat{f}}} {{\hat{g}}},\qquad \widehat{\partial _x f}=i k{{\hat{f}}}, \qquad \widehat{M_\delta }=e^{-\frac{\delta }{2}k^2}, \end{aligned}$$

and obtain an equation for ${{\hat{\rho }}}_g(k,t)=\int e^{-k i x}\rho _g(x,t) \,\mathrm{d}x$.

$$\begin{aligned} \partial _t {{\hat{\rho }}}_g=-i k c_1 {{\hat{\rho }}}_g -\frac{\rho _0}{\zeta }k^2 \left( \mu {{\hat{\rho }}}_g + {{\hat{\phi }}}_k {{\hat{\rho }}}_f\right) . \end{aligned}$$

For the Fourier transform of $\rho _f$, we obtain

$$\begin{aligned} {{\hat{\rho }}}_f(k,t)=\tilde{\delta }(k)-\frac{\gamma }{\eta }\left( 1-e^{-\delta k^2}\right) \hat{\phi }_k {{\hat{\rho }}}_g+\frac{\gamma ^2}{\eta }k^2 {{\hat{\phi }}}_k \partial _t \hat{\rho }_g, \end{aligned}$$

where ${{\tilde{\delta }}}(k)$ is the Dirac delta. Substituting ${{\hat{\rho }}}_f$ into the equation for ${{\hat{\rho }}}_g$ gives

$$\begin{aligned} \partial _t {{\hat{\rho }}}_g(k,t)=\alpha (k){{\hat{\rho }}}_g(k,t), \end{aligned}$$

with $\alpha (k)$ given in (34) as claimed.

(ii) We note that the decay or growth behaviour is determined by the sign of the real part of $\alpha (k)$. Since the denominator will always be positive, it is sufficient to examine the numerator. This gives the result. $\square$

#### Corollary 2

Let $\rho _f$ be given only up to order $\gamma$ and let $\delta \rightarrow 0$. Then the real part of $\alpha (k)$ in Proposition 1 becomes

$$\begin{aligned} \mathfrak {R}\alpha (k) =\frac{\rho _0}{\zeta }k^2 \left( \frac{\gamma }{\eta }(k{{\hat{\phi }}}_k)^2-\mu \right) . \end{aligned}$$

*Interpretation* We interpret the results of Proposition 1(ii) as indication under what conditions patterning is expected. We start by observing that in the absence of obstacle noise, $\delta \rightarrow 0$, the linear stability condition (35) simplifies to

$$\begin{aligned} \max _{k} (k{{\hat{\phi }}}_k)^2<\frac{\mu \eta }{\gamma }. \end{aligned}$$

Since $\frac{1}{\delta }\left( 1-e^{-\delta k^2}\right) \le k^2$, we observe that the obstacle noise $\delta >0$ has a stabilising effect. The constant on the right-hand side is critical for (in)stability. We see that SPP self-repulsion, strong obstacle springs and high obstacle friction stabilise the system. The order $\gamma ^2$ approximation of the obstacle density leads to the additional terms in the denominator. It does not influence whether the constant steady-state destabilises; however, it decreases the growth or decay rate of the perturbations. The main determinant for pattern formation is the SPP–obstacle interaction kernel $\phi$ and the decay behaviour of its Fourier transform or coefficients. In case of purely local interactions, ${{\hat{\phi }}}_k$ is constant and we see that the we have destabilisation for all parameter values, since for large frequencies the real part of $\alpha$ will always become positive. This emphasises the importance of the non-locality of the SPP–obstacle interactions. Next we look at a specific case.

#### Example 1

We assume $$\delta \rightarrow 0$$ and further consider the obstacle density only up to order $$\gamma $$. We work on $$x\in [0,1]$$ with periodic boundary conditions. Further we let the microscopic SPP–obstacle interaction kernel $$\phi $$ be compactly supported on the interval $$[- r_I, r_I]$$ and yield a pushing force that decreases linearly with distance and is continuous at $$ r_I$$, i.e.

$$\begin{aligned} \phi (x)={\left\{ \begin{array}{ll} C \frac{3}{2 r_I}\left( 1-\frac{|x|}{ r_I}\right) ^2 &{}\quad \text{ if } |x|< r_I \\ 0 &{}\quad \text{ else }.\\ \end{array}\right. } \end{aligned}$$

In this case, we can calculate the Fourier coefficients explicitly and obtain

$$\begin{aligned} {{\hat{\phi }}}_k=6 C\frac{ r_I k -\sin {( r_I k)}}{( r_I k)^3}. \end{aligned}$$

The function

$$\begin{aligned} F(k)=(k{{\hat{\phi }}}_k)^2=\left( \frac{6 C}{ r_I}\right) ^2\left( \frac{ r_I k -\sin {( r_I k)}}{( r_I k)^2}\right) ^2 \end{aligned}$$

attains its maximum at $k=\pi / r_I$ and we hence find that if

$$\begin{aligned} \left( \frac{6 C}{\pi r_I}\right) ^2 < \frac{\mu \eta }{\gamma }. \end{aligned}$$

then the spatially constant steady state is linearly stable. The converse is in general not true, since $\pi / r_I$ will typically not be in $2\pi {\mathbb {Z}}$. In the case of destabilisation, we expect the pattern size *P* to be related to the maximum of $\mathfrak {R}\alpha (k)$, given in Corollary 2. We observe that $F(k)\rightarrow 0$ for $k \rightarrow \infty$ and hence $\mathfrak {R}\alpha (k)<0$ for *k* sufficiently large. This means high-frequency perturbations will be damped by the diffusion-like SPP self-repulsion term. Since $\mathfrak {R}\alpha (0)=0$, there will typically be a well-defined maximum attained at some $k=2\pi l_\text {max}$ with $l_\text {max}\in {\mathbb {Z}}$. We then expect that *P* defined by

$$\begin{aligned} P=\frac{1}{l_\text {max}} \end{aligned}$$

will be a good indication of the expected pattern size. We numerically investigate whether this holds also far away from the constant steady state and for the IBM in Sect. 5.2.

### Obstacle–Induced SPP Interaction

In this section, we show how properties of the interactions between SPPs and obstacles on the micro-level inform the properties on the macro level and find some interesting connections to equations for granular flow, porous media and aggregation equations. We focus on the simplest case, where we assume the obstacle noise to be zero and consider the obstacle equation only until order $\gamma$. Further we work in one space dimension where many calculations can be done explicitly. Then the system of interest for the SPP density $\rho _g(x,t)$ and the obstacle density $\rho _f(x,t)$ for $x\in {\mathbb {R}}$ and $t\ge 0$ is given by (17) coupled to (19).

*A Non-local Equation with Gradient Flow Structure* If we substitute $\rho _f$ given in (19) into the equation for $\rho _g$ given in (17), we obtain

36 $$\begin{aligned}&\partial _t\rho _g+c_1\partial _x \rho _g = \frac{1}{\zeta } \partial _x \left[ \rho _g \partial _x \left( \mu \rho _g+ \frac{\gamma }{\eta } \phi ' *\phi '* \rho _g \right) \right] , \end{aligned}$$

We now see that we have a nonlinear, non-local model with a gradient flow structure. These types of equations appear in a wide range of contexts ranging from granular flow, porous media and biological aggregation (Otto 2001; Topaz et al. 2006; Toscani 2000) and their properties are subject of intense study (Ambrosio et al. 2008; Benedetto et al. 1998; Carrillo et al. 2003). The term stemming from the SPP self-repulsion is often written as $\mu \rho =H'(\rho )$, where $H(\rho )=\frac{\mu }{2}\rho ^2$ is the SPP density of internal energy of the system. For the term stemming from the SPP–obstacle interaction, we can define the interaction kernel

37 $$\begin{aligned} W(x)=( \phi ' *\phi ')(x). \end{aligned}$$

Note that while $\phi$ is the microscopic interaction potential between SPPs and obstacles, *W* can be interpreted as macroscopic obstacle–induced SPP interaction potential.

**Fig. 5:**
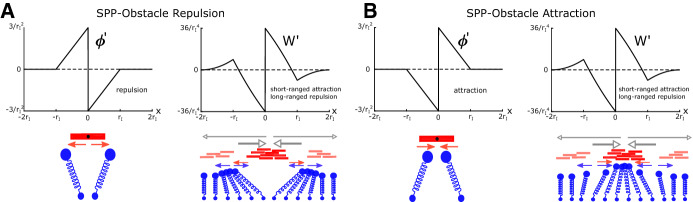
(Color figure online) Micro–macro-interactions. Shown are the microscopic SPP–obstacle interaction force $\phi '(x)$ and the resulting macroscopic interaction force $W'(x)$ for a 1D example, where $\phi (x)$ defined as in Example 1, for (**a**) $C=1$, i.e. repulsive and (**b**) $C=-1$, i.e. attractive. The schematic below illustrates the underlying interactions, where red and blue arrows mark the effect of the SPPs on the obstacles and vice versa, respectively. Grey arrows show the net effect the group of SPPs in the center has on other SPPs

*Bi-phasic Effect at the SPP Level* We now infer properties of *W* (the macro-interaction potential) from properties of $\phi$ (the micro-interaction potential). Note that $\phi '>0$ or $W'>0$ indicate forces to the left and $\phi '<0$ or $W'<0$ indicate forces to the right.

#### Lemma 3

(Obstacle–induced SPP interactions) Let $\phi (x)$ be an even potential. Then $\phi '$ is odd and we can define a function $\varphi$ on $[0,\infty )$ by

38 $$\begin{aligned} \phi '(x)=\varphi (|x|)\,\text {sign}(x), \end{aligned}$$

using the convention that $$\text {sign}(0)=0$$ and defining $$\varphi (0):=\lim _{x\rightarrow 0^+}\varphi (x)$$. Let $$\varphi (0)\ne 0$$ and $$\varphi $$ be continuous with bounded first derivative on $$[0,\infty )$$. We further assume that $$\varphi $$ has compact support on $$[0, r_I]$$ for some $$ r_I>0$$, and that $$\varphi $$ and $$\varphi '$$ have constant but opposite sign on their support. Let *W* be defined as in (37). Then the following holds:

(i) *W* is an even potential continuous on ${\mathbb {R}}$ and continuously differentiable on ${\mathbb {R}}\backslash \{0\}$. *W* has compact support on $[-2 r_I, 2 r_I]$.
(ii) *W* is an attractive potential for short distances, i.e. $W'(x)>0$ for $x>0$, *x* small.
(iii) *W* is a repulsive potential on $( r_I,2 r_I)$, i.e. $W'(x)<0$ for $x\in ( r_I,2 r_I)$.

#### Proof

(i): Since *W* is the convolution of two compactly supported, bounded functions, *W* is continuous. Using the definition of *W* and that $\phi '$ is odd, we calculate

$$\begin{aligned} W(-x)=&\int \phi '(y)\phi '(-x-y)\,\mathrm{d}y\\ =&\int \phi '(-y)\phi '(-x+y)\,\mathrm{d}y = \int \phi '(y)\phi '(x-y)\,\mathrm{d}y=W(x). \end{aligned}$$

Using (38), we calculate $\phi ''(x)=\varphi '(|x|)+2\varphi (0)\delta (x)$, where $\delta$ is the Dirac delta. We therefore obtain

39 $$\begin{aligned} W'(x)=&\,( \phi '' *\phi ')(x)\nonumber \\ =&2\,\varphi (|x|)\varphi (0)\text {sign}(x)+\int \varphi '(|z|)\varphi (|x-z|)\text {sign}(x-z)\,\mathrm{d}z. \end{aligned}$$

The second term is continuous in *x*, since it is the convolution of two compactly supported functions, both bounded, in particular it is zero if evaluated at $x=0$ due to symmetry. The first term is continuous on ${\mathbb {R}}\backslash \{0\}$ hence the same is true for $W'$. That *W* is compactly supported on $[-2 r_I,2 r_I]$ is a consequence of the support of $\phi '$.

(ii): Using (39), we find that

$$\begin{aligned} \lim _{x\rightarrow 0^+}W'(x)=2 (\varphi (0))^2>0, \end{aligned}$$

which together with the results of (i) shows that $W'(x)>0$ for small, but positive *x*. This shows that *W* is an attractive potential for small distances.

(iii): Let $x\in ( r_I, 2 r_I)$. Using (39), we find that

40 $$\begin{aligned} W'(x)=\int _{x- r_I}^{ r_I} \varphi '(z)\varphi (x-z)\,\mathrm{d}z. \end{aligned}$$

By assumption, the product of $$\varphi '$$ and $$\varphi $$ is negative, which shows that *W* is an repulsive potential at distances between $$ r_I$$ and $$2 r_I$$. This finishes the proof. $$\square $$

#### Example 2

(Micro–macro-potentials) We illustrate the results of the above Lemma with two examples of SPP–obstacle potentials. Using the notation introduced in (38), we consider for $r\in [0, \infty )$

$$\begin{aligned} \varphi _1(r)=C \frac{3}{ r_I^2}\left( 1-\frac{r}{ r_I}\right) H( r_I-r),\quad \varphi _2(r)=C \frac{1}{2r_I^2} e^{-r/r_I}, \end{aligned}$$

where *H* is the Heaviside function, $$ r_I>0$$. $$\pm C>0$$ corresponding to attractive and repulsive SPP–obstacle interactions, respectively. The function $$\varphi _1$$ corresponds to the potential of Example 1, which is compactly supported and covered by Lemma 3, while $$\varphi _2$$ corresponds to a kernel without compact support. Figure 5a and b shows the resulting obstacle–induced SPP forces $$W_1'$$ for $$\varphi _1$$ for $$C=-1$$ and $$C=1$$, respectively. For $$\varphi _2$$, we can see the bi-phasic behaviour directly by calculating

$$\begin{aligned} W_2'(x)=\frac{1}{4r_I^5}e^{-\frac{|x|}{r_I}}\left( 2r_I-|x|\right) \text {sign}(x), \end{aligned}$$

showing that $W_2$ is an attractive potential for $|x|<2r_I$ and repulsive otherwise. Note that for both examples the sign of *C* doesn’t affect the shape of $W'$.

Lemma 3 and Example 2 show that the SPP–obstacle interactions will have a short-ranged attractive effect on SPP level, irrespective of whether the micro-interaction was attractive or repulsive. This can be understood intuitively, see Fig. 5: If the SPPs and obstacles repel each other, the obstacles that have been repelled by a group of SPPs will in turn repel other SPPs and therefore lead to further aggregation of the SPPs (Fig. 5a). On the other hand, if the SPPs attract the obstacles, the obstacles attracted by a group of SPPs will attract even more SPPs, again leading to an aggregation effect on the SPP level (Fig. 5b). Further Lemma 3 shows that if the SPP–obstacle interaction force (whether attractive or repulsive) is falling with distance, we see that in addition to the short-ranged attraction, we have a long-ranged repulsion at the SPP level as well. The second example in Example 2 suggests that this property is not limited to compactly supported functions and that Lemma 3 can be generalised to a bigger class to kernels.

These observations already give a good intuition to understand the phenomena observed in Sect. 2.2. For both the moving clusters and the travelling bands, the 1D equations (along the global alignment direction) would correspond to moving aggregates of SPPs. Both the moving clusters and the travelling bands seem to have controlled size, in particular we observed that a cluster that is too big is split into two. The above observations now give an explanation for the observed behaviour: The SPP–obstacle interaction leads to short-ranged SPP attraction and hence aggregation, however, due to the two sources of repulsion (SPP self-repulsion and obstacle–induced repulsion), clusters cannot grow too large. Next we perform 1D simulations to compare the macro-model with the IBM.

## Numerical Results in 1D

In this section, we numerically solve the 1D macro-model for SPP–obstacle interactions and compare the results to both 1D IBM simulations and the analytical results of Sect. 4. Simulation details can be found in “Appendix A.1”.

### Comparing SOH and IBM Simulations

**Fig. 6:**
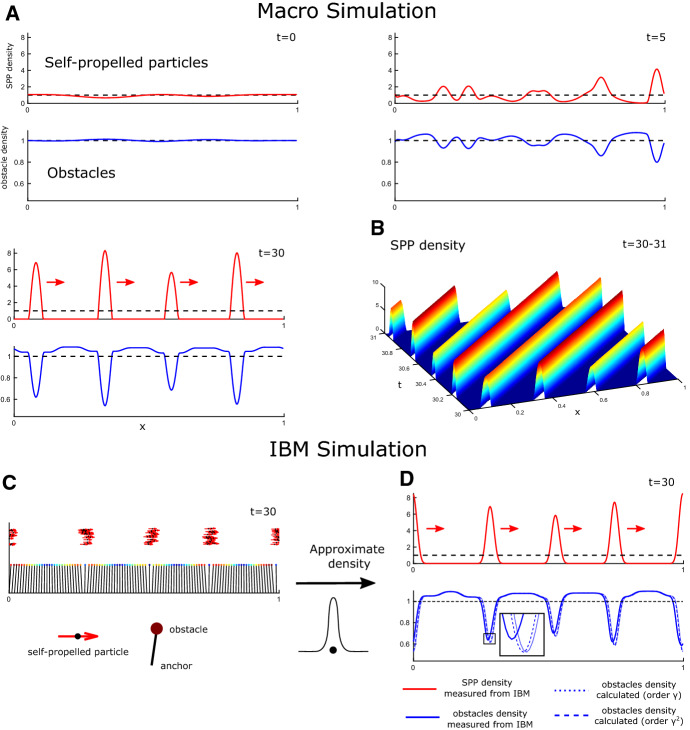
(Color figure online) Simulations of the 1D macro-model and IBM. **a**, **d** Depicted are snapshots of numerical solutions showing the SPP (red, upper rows) and obstacle (blue, lower rows) densities, as well as their (constant) means (dashed black). **a**, **b** Simulations of the macro-model (17), (19). **b** Space–time plot during one time unit of the continuation of the simulation in (**a**). **c**, **d** Simulations of the 1D IBM. **c** Particle *x*-positions of the SPPs (red arrows with black dots) and obstacles (coloured circles, colour indicated displacement), *y*-positions are arbitrary. **d** Approximated and calculated IBM particle densities of the simulation in **c**, see text for details

*The Macro-SPP Obstacle Model Produces Travelling Clusters* We simulate (17) coupled to (19) in 1D using periodic boundary conditions on $$x\in [0,1]$$ and the following parameter choices: $$\eta =1$$, $$c_1=1$$, $$\zeta =8$$, $$\gamma =2\times 10^{-3}$$ and $$\mu =5 \times 10^{-4}$$. We use a linear microscopic interaction force, i.e. the interaction kernel as defined Example 1 with $$ r_I=0.18$$ and $$C=0.25$$. As initial conditions, we use a perturbed uniform SPP density. Figure 6a shows that, indeed, moving clusters of SPPs develop, with stretches of zero density between them. The clusters seem to be relatively evenly spread. The corresponding obstacle density is minimal where the SPP density is maximal. After the clusters have been established, we inspect the space–time plot for one time unit Fig. 6b, which shows that they appear to be stably moving travelling waves of about speed one.

*The Macro-SPP Obstacle Model Agrees with the IBM* Next we compare to 1D IBM simulations of (1). Note that in 1D, we can disregard the orientation equation and assume all particles self-propel to the right. We use the same parameters as for the macro-model with $N=M=100$ and a self-repulsion kernel yielding a linear force, dropping with distance of width $r_R=0.02$. As initial conditions, we use equally spaced anchor points and randomly positioned SPPs. Figure 6c shows the obstacles, their tether points and the SPPs at time $t=30$. We calculate the corresponding SPP and obstacle densities from the particle positions. To that end we create a smoothed version of the empirical distribution defined analogous to (3), where the Dirac delta distributions have been replaced by 1D-Gaussians with variance $1\times 10^{-4}$. Note that choice of the variance is delicate, since it has to be small enough to be able to resolve the patterns and big enough to lead to meaningful averaging. The result is shown in Fig. 6d. A comparison between the simulated SPP and obstacle densities for the macro-model and IBM shows remarkable good agreement both qualitatively and quantitatively.

*Higher-Order Approximations Lead to a Delay Effect* The macro-model was simulated using an order $\gamma$ approximation for the obstacles. To assess the effect of the order $\gamma ^2$ terms without solving the full system, we proceed as follows: We substitute the measured IBM SPP density depicted in Fig. 6d into (18) (with $\delta =0$) to calculate the obstacle density as predicted by the model. We calculate both the order $\gamma$ and order $\gamma ^2$ approximations. For the latter, we need the time derivative of the SPP density, which we approximate by calculating the SPP density at the previous time step and using a forward finite difference approximation. The resulting densities are shown in Fig. 6d. We observe that measured and calculated obstacle densities agree remarkably well. Inspecting the inset in Fig. 6d, we see that the order $\gamma$ approximation predicts the obstacle density minima to be precisely at the SPP density maxima; however, both the order $\gamma ^2$ approximation and the actual measured IBM obstacle density have their local minima shifted backwards with respect to the SPP direction, yielding a better fit between the measured and calculated order $\gamma ^2$ densities that those of the order $\gamma$ approximation. This demonstrates that the derived obstacle equation allows to calculate the obstacle density for a given SPP density. It also shows that the higher-order approximation in $\gamma$ is necessary if one wants to account for effects of SPP movement.

**Fig. 7:**
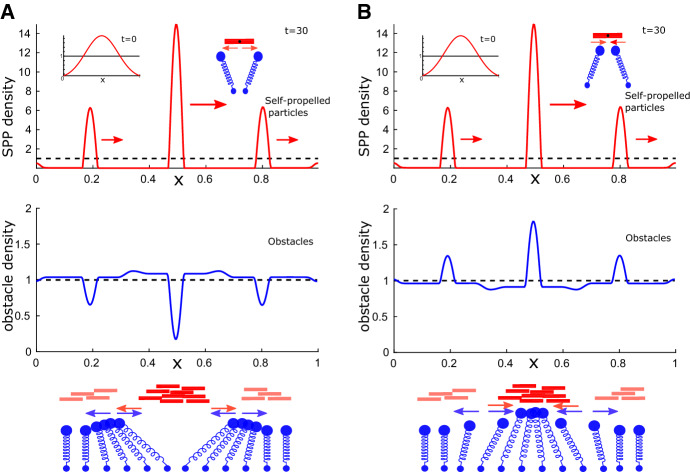
(Color figure online) Simulations of the 1D macro model. **a**, **b** Depicted are snapshots of numerical solutions to (17), (19) showing the SPP (red, upper rows) and obstacle (blue, lower rows) densities, as well as their (constant) means (dashed black). Insets show initial condition, schematic depicts nature of microscopic interaction, red arrows indicate movement direction of the densities. Schematics in lower row: see Fig. 5

### Testing Analytical Insights

*Attractive and Repulsive Interactions Lead to the Same SPP Behaviour* In the next numerical experiment, shown in Fig. 7 we use as initial condition a centrally placed Gaussian and inspect the moving steady state density for a repulsive (A) and an attractive (B) microscopic interaction force. We see that in both cases the resulting SPP density is the same, forming a travelling wave with a stable shape. This shape consists of a large cluster and two smaller clusters to its left and right. However, the obstacle density differs in the two cases: For an attractive potential we have obstacles clusters coinciding with the SPP clusters, whilst for the repulsive potential SPP clusters create regions of low obstacle density. The lower row compares this with the intuitive explanation of the previous section (see Fig. 5).

**Fig. 8:**
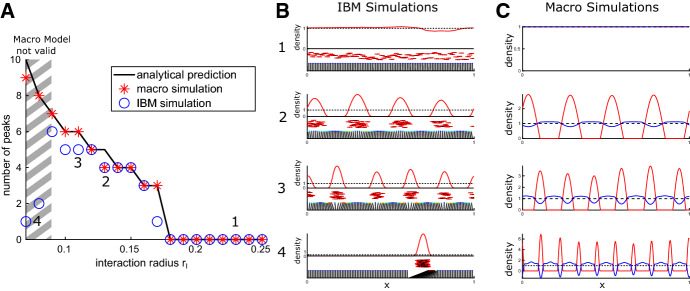
(Color figure online) Linear stability analysis predictions. **a** Number of peaks in dependence of interaction radius $$ r_I$$. Shown are the analytical predictions (black line), the macro-simulation results (red star) and the IBM simulation results (blue circle). Numbers mark the examples in (**b**, **c**). **b**, **c** Final simulation results for the examples marked in A for the IBM (**b**) and the macro-model (**c**). IBM results are depicted as in Fig. 6, macroscopic SPP and obstacle densities are shown in red and blue, respectively

*Linear Stability Analysis Predicts Macro and IBM Patterns* In Sect. 4.1, we performed a linear stability analysis for the 1D macro-equation. In Example 1, we determined the criteria for pattern formation and how to predict pattern size for a specific interaction potential shape. Now we compare these predictions to simulations of both the 1D macro-equations (17), (19) and the 1D IBM simulations by varying the size of the support of the interaction kernel $r_I$. We use the same kernels and number of particles as above and the following parameters: $\eta =1$, $c_1=1$, $\zeta =8$, $\gamma =2\times 10^{-3}$ and $\mu =6.7 \times 10^{-3}$, $C=0.17$. We start with a randomly perturbed constant initial density for the macro-model and regularly spaced anchors and randomly placed SPPs for the IBM. We compare the predicted number of peaks as calculated in Example 1 (and defined as the reciprocal of the pattern size) to the observed number of peaks at time $t=30$. The result is shown in Fig. 8. We find that the analytical predictions of Sect. 4.1 agree very well with the macro model. The agreement with the IBM simulations is good as long as the macro-model gives physically meaningful (i.e. positive) obstacle densities (examples 1, 2, 3 in Fig. 8), but breaks down otherwise (example 4 in Fig. 8). This shows both that the macro-model can be used to gain insights into the IBM, but also that it is limited to certain parameter regimes.

## Discussion

*Summary* In this work, we formulated an IBM model of the interaction of self-propelled, collectively moving SPPs with elastically tethered obstacles. Despite the seemingly simplicity of the interactions, we found that the system can self-organise into a big variety of patterns, including travelling bands, (transiently stable) trails and size-controlled clusters. To investigate these patterns further, we derived macroscopic equations for the obstacle and SPP densities and the SPP orientation. The asymptotic regime of interest assumed $\gamma$ to be small, i.e. fast obstacle spring relaxation (strong obstacle springs). The resulting continuum equations are nonlinear and contain a non-local interaction term. Linear stability analysis allowed to estimate pattern size from model parameters and showed which effects promote pattern formation (e.g. obstacle–SPP interaction) and which stabilise the unpatterned state (e.g. SPP self-repulsion). We found that, surprisingly, SPP dynamics are independent of whether obstacles and SPPs repel or attract each other. In particular, in Sect. 4 we discovered that the macroscopic SPP equation has gradient flow structure with a bi-phasic (short-range attractive, long-range repulsive) non-local obstacle–induced interaction kernel.

*Biological Implications* SPPs represent moving individuals such as animals, pathogens, bacteria, sperm, cancer or other cells. The elastically tethered obstacles mimic a complex environment, which acts on and reacts to SPPs, e.g. by repulsion. They represent, e.g. a fibrous network which is relatively fixed in space, but whose components can be pushed upon SPP interaction, after which they relax back to their original positions. Simulations revealed several patterns, such as moving clusters, where tightly packed groups of cells move together. This is a commonly observed phenomena, e.g. for groups of cancer cells invading a tissue. The key observation is that the model does not include any explicit SPP attraction, making the formation of high density patterns surprising. The subsequent analysis of the macromodel (derived in Theorem 1) gives a quantitative explanation for the observed behaviour: We found that both attractive and repulsive microscopic interactions between SPPs and obstacles cause a short-range attractive macroscopic effect on the SPP level, which leads to clustering. Clustering of organisms is ubiquitous in nature and is often attributed to direct attraction between the individuals. However, our results suggest that the apparent attraction could be indirect and is in fact mediated by the environment. In other words, it is possible the individuals feel no attraction towards each other, but will still form tight clusters. This finding is highly relevant to understanding cell clustering or swarm formation. The analysis in Proposition 1 allows to assess the formation and size of patterns (such as moving clusters or travelling bands), without the need to simulate. A key finding is that the interaction strength between SPPs and obstacles has to be large compared to the SPP self-repulsion and the tethering strength in order to create patterns. This means that, e.g. cell clustering, could be promoted by a more elastic environment. An important biological implication is that one can influence cell aggregation by modifying only the environment.

*Future Work* The 1D model examined in Sects. 4 and 5 cannot describe trail patterns and does not allow to distinguish between clusters and travelling bands. In the future, we plan to extend both the simulations and the analysis of the continuum model to two- and three-space dimensions. In particular, we expect new insights about pattern directionality from a linear stability analysis in higher dimensions. Our derivation relied heavily on the assumptions of smallness of $\gamma$. Mathematically this limitation manifests in the fact that the obstacle density can become negative, at which point the model becomes invalid. In the future, we would like to derive macroscopic models that are and remain well-posed for any parameter combination. This will require a different closure method of the kinetic equations. Our current model seems to be able to capture several of the observed phenomena at the IBM model, such as the travelling bands or the clusters, however, for example the trail formation pattern will most likely require an extension of the current techniques. We also plan further analysis of the continuum model, capitalising on its gradient-flow structure. Strong analytical results, such as energy dissipation estimates, exist for these type of equations, which suggests that it is possible, at least for certain cases, to construct steady states and assess their stability in a rigorous manner. Obvious extensions include 1D simulations of the SPP–obstacle model using an ${\mathcal {O}}(\gamma ^2)$ approximation of the obstacle density or including the positional noise.

The current model describes interactions between SPPs and obstacles. In many instances, however, all components are immersed in a fluid. Past work has already studied how to derive and analyse SPP–fluid interactions (Degond et al. 2019). There exist models for how fluid properties are affected if it contains immersed objects. A famous example is the Oldroyd-B model, describing the visco-elasticity of fluids filled with spring dumbbells (Oldroyd 1950). We plan to use our derivation strategy to derive equations for fluids filled with tethered obstacles and study how fluid properties such as viscosity are affected. An additional level of complexity we plan to tackle, is to combine all three components, the fluid, the obstacles and the SPPs. In this case a natural question appears: How big are the obstacles compared to the SPPs. The flexible techniques developed in this work will allow to answer this question by performing the coarse-graining at different levels.

Applying the findings to biological systems, such as sperm movement, will require careful parametrisation of the model. An advantage of the IBM formulation is that model parameters are relatively easy to obtain from experiments: Diffusion constant can be estimated using the Stokes–Einstein formula and interaction radii can be linked to object sizes. Other key properties, such as how forces behave with respect to distance, could be measured by observing how an individual SPP reacts to an individual obstacle. The model then allows to predict the result of the dynamics of large groups of SPPs and obstacles.

## Supplementary Material

*IBM Simulation Videos* The three supplementary videos

- moving_clusters.avi
- trails.avi
- travelling_bands.avi

show the dynamics in time of the 2D IBM simulations depicted in Fig. 2. SPPs are shown in red, obstacles in blue.

### Electronic supplementary material

Below is the link to the electronic supplementary material.

Supplementary material 1 (avi 39412 KB)

Supplementary material 2 (avi 28519 KB)

Supplementary material 3 (avi 50347 KB)

## Data Availability

No new data were collected in the course of this research.

## Appendix

### Simulation Details

*IBM Simulations of Sect.* 2.2 We simulate the IBM model (1) in two space dimensions using MATLAB with a timestep of $\varDelta t=10^{-3}$. Model parameters are listed in Sect. 2.2. Numerically we use the circle method described in Motsch and Navoret (2011).

*Macro-model Simulations in 1D of Sect.* 5: We simulation the macroscopic model (17), (19) in one space dimension using MATLAB with spatial and temporal timesteps of $\varDelta x=3\times 10^{-3}$, $\varDelta t=10^{-2}$. The method used is described in Carrillo et al. (2015).

### Properties of the Operator ${\mathcal {B}}$ Defined in (27)

It is a well-known fact that the operator ${\mathcal {B}}$ defined in (27) is the generator of the Ornstein–Uhlenbeck stochastic process (see Pavliotis 2014). For an extensive study of this operator, we refer the reader to Achleitner et al. (2015), Risken (1996), or Reed and Simon (1978). In the following result, we collect a few properties needed in this paper.

#### Lemma 4

(Properties of ${\mathcal {B}}$) Let the operator ${\mathcal {B}}$ be defined by (27) and let $\varvec{i}=(i_1,i_2,i_3)$ be a multi-index. We define

$$\begin{aligned} {\mathcal {H}}_{\varvec{i}}(\sigma )=H_{i_1}(\sigma _1)H_{i_2}(\sigma _2)H_{i_3}(\sigma _3), \end{aligned}$$

where

$$\begin{aligned} H_j(s)= (-1)^j e^{\frac{s^2}{2}}\frac{\,\mathrm{d}^j}{\,\mathrm{d}s^j} e^{-\frac{s^2}{2}}. \end{aligned}$$

Note that $H_j(s)$ are the (probabilistic) Hermite polynomials. Let us consider the following $L^2$-weighted space

$$\begin{aligned} X := \bigg \{ f \in L^2( {\mathbb {R}}^3) : \int _{{\mathbb {R}}^3} f^2 M_1 \,\mathrm{d}\sigma < \infty \bigg \} \, , \end{aligned}$$

where $M_1$ is defined in (12) taking $\delta = 1$. For any two functions *f* and *g* in *X*, we define their weighted inner product by

$$\begin{aligned} \langle f, g \rangle _X := \int _{{\mathbb {R}}^3} f(\sigma ) g(\sigma )M_1(\sigma ) \,\mathrm{d}\sigma . \end{aligned}$$

We then have the following properties:

1. $\langle {\mathcal {H}}_{\varvec{i}}, {\mathcal {H}}_{\varvec{j}} \rangle _X = \varvec{i}! \delta _{\varvec{i}\varvec{j}}$, where $\delta _{\varvec{i}\varvec{j}}$ is the Kronecker delta for multi-indices.
2. $${\mathcal {B}}({\mathcal {H}}_{\varvec{i}})=-|\varvec{i}|{\mathcal {H}}_{\varvec{i}}$$.
3. The set $\{{\mathcal {H}}_{\varvec{i}}\}_{\varvec{i}}$ is a complete orthogonal basis of the $L^2$-weighted space *X*.
4. $${\mathcal {H}}_{e_i}{\mathcal {H}}_{e_k}={\mathcal {H}}_{e_i+e_k}+\delta _{ik}{\mathcal {H}}_{\varvec{0}}$$.
5. $${\mathcal {H}}_{e_i}{\mathcal {H}}_{e_j + e_k}={\mathcal {H}}_{e_i+e_j+e_k}+\delta _{ik}{\mathcal {H}}_{e_j}+\delta _{ij}{\mathcal {H}}_{e_k}$$.

We have used the notation $\varvec{i}!=i_1!i_2!i_3!$ and $|\varvec{i}| = i_1+i_2+i_3$. Note that P1 shows that ${\mathcal {H}}_{\varvec{i}}$ are orthogonal with respect to the inner product $\langle \cdot , \cdot \rangle _X$ and P2 states that ${\mathcal {H}}_{\varvec{i}}$ are eigenfunctions with eigenvalue $-|\varvec{i}|$. In the product rules P4 and P5, $e_i$ denotes the *i*-th unit vector in ${\mathbb {R}}^3$.

### Calculation of the Obstacle Density

In this section, we detail the calculations of 0-th order moment of *f*, $\rho _f$, in terms of expansions with respect to $\gamma$ and $\delta$. As outlined in the main text, we will perform the following steps:

1. Perform the change of variables $\sqrt{\delta }\sigma =x-y$. This changes the integrand to be proportional to $M_1(\sigma )h_i(\sigma , x-\sqrt{\delta }\sigma ,t)$ for $\rho _{f_i}$.
2. Next we Taylor expand $h_i(\sigma ,x-\sqrt{\delta }\sigma ,t)$ around $\delta =0$ using the expansions of above.
3. Then we calculate the contributions using the scaling condition (31), the orthogonality of the Hermite polynomials (P1) and the product rule (P4) of Lemma 4.

The following result will be helpful for the subsequent calculations.

#### Lemma 5

Let $h_1^k$ and $h_2^k$, for $k = 0, 1, \ldots$, be the solutions of the above expansion and let their representations w.r.t the basis of Hermite polynomials be given by

$$\begin{aligned} h_1^k=\sum _{\varvec{i}} a^k_{\varvec{i}} {\mathcal {H}}_{\varvec{i}}(\sigma ), \qquad h_2^k=\sum _{\varvec{i}} b^k_{\varvec{i}} {\mathcal {H}}_{\varvec{i}}(\sigma ), \end{aligned}$$

where $\varvec{i}$ is a multiindex and $a^k_{\varvec{i}}$ and $b^k_{\varvec{i}}$ are functions of *y* and *t*. Then it holds that

$$\begin{aligned} a^k_{\varvec{i}}&\equiv 0 \qquad {\mathrm{for}} \quad |\varvec{i}|\!\!\! \mod 2 = k \!\!\!\mod 2, \quad {\mathrm{or}} \quad |\varvec{i}|>k+1\\ b^k_{\varvec{i}}&\equiv 0 \qquad {\mathrm{for}} \quad |\varvec{i}| \!\!\!\mod 2 \ne k \!\!\!\mod 2, \quad {\mathrm{or}} \quad |\varvec{i}|>k+2 \end{aligned}$$

#### Proof

This can be shown by induction. For the initial case, we use the explicit solutions given in (29) and (30). $\square$

**Notation:** In the following, if no further argument is given, functions are evaluated at $(\sigma ,x,t)$ and $\partial _i:=\frac{\partial }{\partial x_i}$. We use the Einstein summation convention. In general, we often suppress the dependence on time *t*.

#### Remark 5

In the following, we often use Lemma 5 in combination with the fact that odd-order moments of $M_1$ are zero.

Preparation for Step 2 in the above procedure: Taylor expand $h_r(\sigma ,x-\sqrt{\delta }\sigma ,t)$,

41 $$\begin{aligned} h_r(\sigma , x-\sqrt{\delta }\sigma )=&h_r^0-\sqrt{\delta }\sigma _k \partial _k h_r^0+\sqrt{\delta }h_r^1\nonumber \\&+\frac{\delta }{2}\sigma _i\sigma _j\partial _{ij}h_r^0-\delta \sigma _i \partial _i h_r^1 + \delta h_r^2 + {\mathcal {O}}(\delta ^{3/2}), \end{aligned}$$

where $r = 1, 2$. We start with the **0-th order density** $\rho _{f_0}$:

$$\begin{aligned} \rho _{f_0}(x,t)=\int f_0(x,y)\,\mathrm{d}y= \int M_\delta (x-y) \,\mathrm{d}y=1 \end{aligned}$$

For the **first order density** $\rho _{f_1}$, we use the reformulation in terms of $h_1$:

$$\begin{aligned} \rho _{f_1}(x,t)&=\int f_1(x,y)\,\mathrm{d}y=\frac{1}{\sqrt{\delta }} \int M_\delta (x-y) h_1\left( \frac{x-y}{\sqrt{\delta }},y\right) \,\mathrm{d}y\\&=\frac{1}{\sqrt{\delta }} \int M_1(\sigma ) h_1\left( \sigma ,x-\sqrt{\delta }\sigma \right) \,\mathrm{d}\sigma \\&=\langle 1, -\sigma _k \partial _k h_1^0\rangle + {\mathcal {O}}(\delta ) \end{aligned}$$

In the second line, we have used Step 1, the change of variables. In the third line, we have used (41) for $r=1$ together with the fact that the order $\delta ^{-1/2}$ term and the order 1 term involving $h_1^1$ are zero due to the normalisation condition (31). For the order $\sqrt{\delta }$-terms we used Remark 5 to show it is zero.

Hence we are left with one term. We use (29) and calculate

$$\begin{aligned} \langle 1, -\sigma _k \partial _k h_1^0\rangle&=- \partial _k {{\tilde{v}}}_i\langle {\mathcal {H}}_{e_k}, {\mathcal {H}}_{e_i}\rangle =- \partial _k {{\tilde{v}}}_k, \end{aligned}$$

where we have used P1 of Lemma 4, i.e. $\langle {\mathcal {H}}_{e_k},{\mathcal {H}}_{e_i}\rangle =\delta _{ki}$. This shows that indeed

$$\begin{aligned} \rho _{f_1}(x,t)&=- \partial _k {{\tilde{v}}}_k+ {\mathcal {O}}(\delta ) \end{aligned}$$

and finishes the calculations for $\rho _{f_1}$. The calculations for the order $\delta$ term are similar and omitted here.

We continue in similar fashion with the **second order density** $\rho _{f_2}$: We use the reformulation in terms of $h_2$ and get

$$\begin{aligned} \rho _{f_2}(x,t)&=\int f_2(x,y)\,\mathrm{d}y=\frac{1}{\delta } \int M_\delta (x-y) h_2\left( \frac{x-y}{\sqrt{\delta }},y\right) \,\mathrm{d}y\\&=\frac{1}{\delta } \int M_1(\sigma ) h_2\left( \sigma ,x-\sqrt{\delta }\sigma \right) \,\mathrm{d}\sigma . \end{aligned}$$

If we now inspect (41) for $r=2$, we find that, as above the scaling condition (31) leads to the $\delta ^{-1}$ order term involving $h_2^0$, the $\delta ^{-1/2}$-order term involving $h_2^1$ and the order one term involving $h_2^2$ being zero. For the remaining $\delta ^{-1/2}$-order term we refer to Remark 5 and hence it is also 0. For the remaining order one terms we calculate

$$\begin{aligned} A_1&:=\frac{1}{2} \int M_1(\sigma ) \sigma _i\sigma _j \partial _{ij}h_2^0 \,\mathrm{d}\sigma =\frac{1}{4} \partial _{ij}({{\tilde{v}}}_k{{\tilde{v}}}_l)\int M_1(\sigma ) \sigma _i\sigma _j {\mathcal {H}}_{e_k+e_l} \,\mathrm{d}\sigma \\&=\frac{1}{4} \partial _{ij}({{\tilde{v}}}_k{{\tilde{v}}}_l)\langle {\mathcal {H}}_{e_i+e_j}, {\mathcal {H}}_{e_k+e_l}\rangle \, ,\\ A_2&:=- \int M_1(\sigma ) \sigma _i \partial _{i}h_2^1 \,\mathrm{d}\sigma =\partial _i(\partial _t {{\tilde{v}}}_k-{{\tilde{v}}}_j \partial _j {{\tilde{v}}}_k) \int M_1(\sigma ) \sigma _i {\mathcal {H}}_{e_k}\,\mathrm{d}\sigma \\&=\partial _i(\partial _t {{\tilde{v}}}_k-{{\tilde{v}}}_j \partial _j {{\tilde{v}}}_k) \langle {\mathcal {H}}_{e_i}, {\mathcal {H}}_{e_k}\rangle \, , \end{aligned}$$

where we have used P1 and P4 of Lemma 4. We continue

$$\begin{aligned} A_1&= \frac{1}{2}\partial _{ij}({{\tilde{v}}}_i{{\tilde{v}}}_j) \, ,\\ A_2&= \partial _i(\partial _t {{\tilde{v}}}_i-{{\tilde{v}}}_j \partial _j {{\tilde{v}}}_i) \, , \end{aligned}$$

where we used the identity

$$\begin{aligned} \langle {\mathcal {H}}_{e_i+e_j}, {\mathcal {H}}_{e_k+e_l}\rangle =\delta _{ik}\delta _{jl}+\delta _{il}\delta _{jk} \end{aligned}$$

for $A_1$. Finally we calculate

$$\begin{aligned} \rho _{f_2}(x,t)&=A_1+A_2+{\mathcal {O}}(\delta )=\left\{ \partial _t \partial _i {{\tilde{v}}}_i+\frac{1}{2}\partial _i\left[ {{\tilde{v}}}_i \partial _j {{\tilde{v}}}_j-{{\tilde{v}}}_j\partial _j {{\tilde{v}}}_i\right] \right\} +{\mathcal {O}}(\delta ). \end{aligned}$$

Note the fact that the remaining term is ${\mathcal {O}}(\delta )$ and not ${\mathcal {O}}(\sqrt{\delta })$ is again thanks to Remark 5 and does not require explicit knowledge of the shape of $h_2^3$.
